# Effectiveness of Web-Based Tailored Advice on Parents’ Child Safety Behaviors: Randomized Controlled Trial

**DOI:** 10.2196/jmir.2521

**Published:** 2014-01-24

**Authors:** Mirjam Elisabeth Johanna van Beelen, Tinneke Monique Jozef Beirens, Paul den Hertog, Eduard Ferdinand van Beeck, Hein Raat

**Affiliations:** ^1^Erasmus MC – University Medical Centre RotterdamDepartment of Public HealthRotterdamNetherlands; ^2^Dutch Association for Youth Health Care PhysiciansUtrechtNetherlands; ^3^Consumer Safety InstituteAmsterdamNetherlands

**Keywords:** child, eHealth, injury, parent, prevention, primary care, RCT, safety

## Abstract

**Background:**

Injuries at home are a major cause of death, disability, and loss of quality of life among young children. Despite current safety education, required safety behavior of parents is often lacking. To prevent various childhood disorders, the application of Web-based tools has increased the effectiveness of health promotion efforts. Therefore, an intervention with Web-based, tailored, safety advice combined with personal counseling (E-Health4Uth home safety) was developed and applied.

**Objective:**

To evaluate the effect of E-Health4Uth home safety on parents’ safety behaviors with regard to the prevention of falls, poisoning, drowning, and burns.

**Methods:**

A randomized controlled trial was conducted (2009-2011) among parents visiting well-baby clinics in the Netherlands. Parents were randomly assigned to the intervention group (E-Health4Uth home safety intervention) or to the control condition consisting of usual care. Parents in the intervention condition completed a Web-based safety behavior assessment questionnaire; the resulting tailored safety advice was discussed with their child health care professional at a well-baby visit (age approximately 11 months). Parents in the control condition received counseling using generic safety information leaflets at this well-baby visit. Parents’ child safety behaviors were derived from self-report questionnaires at baseline (age 7 months) and at follow-up (age 17 months). Each specific safety behavior was classified as safe/unsafe and a total risk score was calculated. Logistic and linear regression analyses were used to reveal differences in safety behavior between the intervention and the control condition at follow-up.

**Results:**

A total of 1292 parents (response rate 44.79%) were analyzed. At follow-up, parents in the intervention condition (n=643) showed significantly less unsafe behavior compared to parents in the control condition (n=649): top of staircase (23.91% vs 32.19%; OR 0.65, 95% CI 0.50-0.85); bottom of staircase (63.53% vs 71.94%; OR 0.69, 95% CI 0.53-0.88); top and bottom of staircase (68.94% vs 78.28%; OR 0.62, 95% CI 0.48-0.81); storage of cleaning products (30.33% vs 39.91%; OR 0.67, 95% CI 0.53-0.85); bathing of the child (23.46% vs 32.25%; OR 0.65, 95% CI 0.51-0.84); drinking hot fluids (34.84% vs 41.73%; OR 0.76, 95% CI 0.61-0.96); using rear hotplates (79.34% vs 85.27%; OR 0.67, 95% CI 0.50-0.90); and the total risk score in which a higher score indicates more unsafe behavior (mean 13.63, SD 6.12 vs mean 15.34, SD 6.07; beta –1.59, 95% CI –2.26 to –0.93). There were no significant differences for other specific behaviors between the two study conditions.

**Conclusions:**

Compared to generic written materials, the E-Health4Uth home safety intervention seems more effective in promoting parents’ safety behavior for safe staircases, storage of cleaning products, bathing, drinking hot fluids, and cooking. This study supports the application of Web-based, tailored, safety advice for the prevention of unintentional injuries in the youth health care setting.

**Trial Registration:**

Nederlands Trial Register: NTR1836; http://www.trialregister.nl/trialreg/admin/rctview.asp?TC=1836 (Archived by WebCite at http://www.webcitation.org/6MPIGQxpx).

## Introduction

### Background

Unintentional injuries are a major cause of death, and a major source of morbidity and loss of quality of life among children aged 0-4 years [1-4]. In children aged 5 years or younger, more than 90% of unintentional injuries occur in and around the home [1]. Although the type and cause of injury varies by age, the most common injuries of children aged 0 to 4 years are falls, poisoning, drowning, and burns [1,5]. Each year in the Netherlands, 18 children aged between 0 and 4 years die because of injuries in and/or around the home [6]. Moreover, an additional 46,000 children aged between 0 and 4 years are medically treated because of home injuries [6]. To reduce the number of injuries, the Dutch Consumer Safety Institute introduced the use of safety information leaflets at preventive youth health care centers to provide safety education to parents of children aged between 0 and 4 years [7]. These leaflets are successfully employed in preventive youth health care and appear to have a modest effect on parental behavior [8,9]. Many countries have installed preventive youth health care, which refers to various activities to improve and protect the health, growth, and development of young people, and to prevent illness and disability in early life. These activities include a system of maternal and child health care, which serves children from birth to age 18 years [10,11]. The preventive youth health care also plays a significant role in injury prevention [12].

In the Netherlands, all parents are invited to regularly attend (free of charge) scheduled well-child visits at their well-baby clinic. During these visits, the growth and development of the child is monitored and relevant health information and vaccinations are provided. In the Netherlands, approximately 93% of parents attend 1 or more well-baby visits when their child is aged 4 years and younger; the attendance rates range from approximately 50% to 93% between the specific age-related scheduled visits [13]. Parents receive health information on various topics, including nutrition, growth, and child home safety [14]. Currently, this safety information is provided to parents by using generic information leaflets that they receive at their regular visits to the well-baby clinic. Nevertheless, the required safety behavior of parents is often lacking, causing unnecessary risk of injury to young children [15-17].

To prevent other childhood disorders, the application of Web-based tailored tools (eHealth) has increased the effectiveness of health promotion effects [18-20]. The field of eHealth, health services and information delivered or enhanced through the Internet and related technologies [21], is broad and emerging at the intersection of medical informatics, public health, and business. It involves the use of information and communications (especially the Internet) to improve or enable health and health care [22]. It could also be used to provide information to parents on several health topics, including home safety. Because tailored information combined with counseling, which can be provided by using eHealth, is personalized, parents could find the information more useful than general information materials [23]. Furthermore, parents may be more inclined to change their behavior when the information they receive is perceived as personally relevant [24,25].

A home safety intervention with Web-based, tailored, safety information was developed and applied (E-health4Uth). It uses Web-based, tailored, safety information in combination with personal counseling at well-baby clinics for safety behaviors required with a child at home. A pilot study showed that most parents found this new safety information to be useful and applicable, and that child health care professionals were enthusiastic about the eHealth intervention [26]. However, no information is available about the effects of the new Internet-based, tailored, safety information on parents’ child safety behaviors compared to the older method of safety education. Tailored information is thought to promote behavior change by providing personally relevant feedback. Tailoring is defined as “any combination of information or change strategies intended to reach one specific person, based on characteristics that are unique to that person, related to the outcome of interest, and have been derived from an individual assessment” [27]. Although (online) computer-tailored interventions seem to have a positive effect on adult behaviors compared to generic information or to no information [17,28], there is no evidence for the efficacy of a tailored intervention on parents’ specific child safety behaviors for the prevention of unintentional injuries.

### Objective of the Study

This study evaluates the effect of Web-based, tailored, safety information combined with personal counseling on parents’ child safety behaviors for the prevention of falls, poisoning, drowning, and burns. The hypothesis is that parents in the E-Health4Uth home safety intervention condition will show less unsafe behavior and will have a lower total risk score 6 months postintervention compared to parents in the control condition with usual care. In addition, the use and application of the E-Health4Uth home safety module and the well-baby visit, including the use of the tailored safety advice, will be evaluated.

## Methods

### Study Design

The E-Health4Uth home safety study (BeSAFE study) is a randomized controlled trial (NTR1836) with a baseline measure point before the intervention and a follow-up measure point 6 months after the intervention; the study is described in detail elsewhere [29]. The Medical Ethics Committee of the Erasmus Medical Center gave a declaration of no objection for this study (MEC-2008-370).

### Participants and Procedures

#### Overview

**Figure 1:**
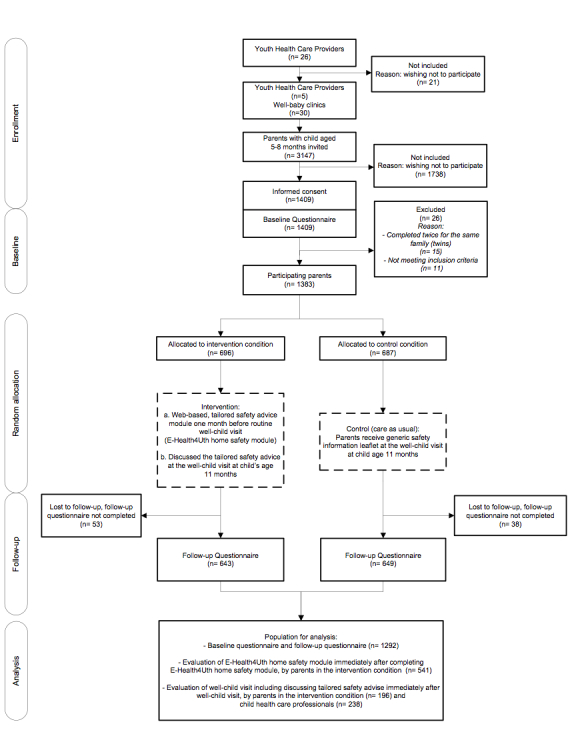
Flow diagram of the enrollment and follow-up of study participants.

A flow diagram of the enrollment and follow-up of study participants is schematically described in Figure 1.

Managers of an opportunity sample of 26 youth health care organizations in the Netherlands were informed about the study and invited to participate. A total of 5 youth health care organizations in the mixed urban-rural provinces of Zuid-Holland, Noord-Brabant, and Zeeland volunteered to participate, with a total of 30 well-baby clinics.

All parents with a child aged between 5 and 8 months (1 parent per family) who were eligible for a routine well-baby visit at their well-baby clinic from June 2009 until December 2010 received written information about the study and were invited to provide informed consent to participate (n=3147). Parents who provided informed consent were invited to complete the baseline questionnaire.

Subsequently, parents were randomly assigned to one of two conditions: (1) Web-based, tailored, safety advice module combined with discussion of the tailored safety advice at the well-baby visit (E-Health4Uth home safety intervention condition), or (2) care as usual (ie, received a generic written safety information leaflet at the well-baby visit; control condition). Randomization was done using a computerized random allocation generator.

Parents in the intervention condition were invited to complete the E-Health4Uth home safety module when their child was approximately 10 months old (ie, 1 month before their routine well-baby visit at the well-baby clinic). The intervention is described in detail in the following section. Parents in the control condition also visited their well-baby clinic when their child was approximately 11 months of age (see control condition described subsequently). All parents received a follow-up questionnaire when their child was approximately 17 months (6 months postintervention). The baseline and follow-up data were collected from June 2009 until July 2011.

Parents received a maximum of 2 regular mail reminders for completing the questionnaires. Parents who did not respond to the invitations to complete the follow-up questionnaire received a telephone call to motivate them to complete the intervention or the questionnaire. Parents in the intervention condition received a maximum of 2 reminders to complete the E-Health4Uth home safety module. If they did not respond, they received a telephone call to motivate them to complete the E-Health4Uth home safety module.

Parents in both conditions who completed the baseline questionnaire received a gift voucher of €15. Parents who also completed the follow-up questionnaire received a second gift voucher of €10.

#### E-Health4Uth Home Safety Intervention Condition

The E-Health4Uth home safety intervention aims at 4 major topics with regard to safety in/around the home of children aged between 12 and 24 months: prevention of falls, poisoning, drowning, and burns [7,8,17,30-34]. The components of the 4 safety topics of the intervention are shown in Multimedia Appendix 1.

Parents allocated to the E-Health4Uth home safety intervention condition received a personal log-in name and password by email when their child was approximately 10 months. Parents were asked to complete the E-Health4Uth home safety module before their next routine well-baby visit at approximately 11 months of age. Parents could complete the E-Health4Uth home safety module wherever they wished (eg, at home, at work) as long as Internet was available. As a first step, parents completed a safety assessment questionnaire. The answers to this assessment questionnaire were used to generate tailored safety advice, which parents could read immediately online. The tailored safety advice was personalized with the child’s name and consisted of messages tailored to the parent’s current situation and safety behavior (Multimedia Appendices 2-4). This included sections with general information on the importance and relevance of the injury area. A total of 114 messages were developed for this tailored safety advice, which could be combined in various ways based on the parent’s answers to the assessment questionnaire.

When parents had completed reading their personal safety advice, they were invited to formulate an implementation-intention plan. In this implementation-intention plan, parents planned specific actions (ie, what, when, and where to improve their safety behavior and implement these in their home situation at a specified time) [35,36].

The tailored safety advice and implementation-intention plan of each parent was sent by email to both the parent and the child health care professional to prepare for the routine well-baby visit at age 11 months. At the well-baby visit, the child health care professional discussed the tailored safety advice and the implementation-intention plan with the parent using motivational interviewing techniques [35-37]. Preceding the start of the study, health care professionals received training from the researchers. In this training, the study was explained and examples of the tailored safety advice were used to give instructions on how the intervention should be delivered to ensure integrity of delivery of the tailored safety advice.

Parents in the intervention condition received the E-Health4Uth home safety intervention, but could also receive the generic safety information leaflet as a part of usual care.

Approximately 4 weeks after the well-baby visit, parents received a reminder about their tailored safety advice and the implementation-intention plan by email to strengthen the message.

The content and development of the E-Health4Uth home safety module was not changed during the study. The intervention software (TailorBuilder) was developed by OverNite Software Europe (OSE, Sittard, the Netherlands).

#### Control Condition

Parents in the control condition received care as usual; that is, parents received a generic safety information leaflet (for children aged 12-24 months) published by the Dutch Consumer Safety Institute [7,9,10,34] during their routine well-baby visit at approximately 11 months of age. During this well-baby visit, the child health care professional discussed the safety in and around the home with the parents using the generic safety information leaflet and motivated parents to adopt safety measures in their home.

The safety information leaflet contained relevant information on the prevention of toddler injuries in and/or around the home, such as information on safety and advice about the prevention of falls (ie, window protection, stair gates, practice walking down the stairs), poisoning (ie, safe storage of cleaning products and medicines), drowning (ie, ponds), and burns (ie, hot fluids, hot pans) [7].

### Outcomes/Measures

#### Overview

Data on demographic factors and parents’ child safety behaviors were collected at enrollment at approximately age 7 months (baseline) and at 6 months postintervention at approximately age 17 months (follow-up) by self-report questionnaires.

#### Parents’ Child Safety Behaviors

In the two study conditions, specific parents’ child safety behaviors for the prevention of falls, poisoning, drowning, and burns were assessed. Some behaviors were assessed only when they were applicable to the situation of the parent. Each specific safety behavior was first classified as being safe or unsafe. Both the E-Health4Uth home safety intervention and the generic safety information leaflet covered the same topics with regard to the prevention of falls, burns, drowning, and poisoning.

Prevention of falls was assessed by the safety of staircases (only if a staircase was present) and window safety (only if there was a window a child could reach present). Safety of staircases was assessed with 4 items: presence of a stair gate at the top of the stairs (yes/no); closing a stair gate at the top of the stairs (always/often/sometimes/rarely/never); presence of a stair gate at the bottom of the stairs (yes/no); and closing a stair gate at the bottom of the stairs (always/often/sometimes/rarely/never). Safe behavior for the top of the staircase was defined as having a stair gate at the top of the staircase and always closing the stair gate. Safe behavior for the bottom of the staircase was defined as having a stair gate at the bottom of the staircase and always closing the stair gate. Additionally, safe behavior regarding the top/bottom of the staircase was defined as having a stair gate at the top/bottom of the staircase and always closing the stair gates. Safety of windows was assessed with 1 item: having window guards on windows a child can reach (yes/no). Safe behavior was defined as having a window guard on windows a child can reach.

Prevention of poisonings was assessed by the storage of cleaning products and medicines. Storage of cleaning products was assessed with 12 items: presence of cleaning products at different storage locations (yes/no). Safe storage of cleaning products was defined as storing them on a shelf or in a drawer or cabinet without a lock higher than 1.50 m or storing them in a drawer or cabinet with a lock. Storage of medicines was assessed with 13 items: presence of medicines at different storage locations (yes/no). Safe storage of medicines was defined as storing them on a shelf or in a drawer or cabinet without a lock higher than 1.50 m or storing them in a drawer or cabinet with a lock.

Prevention of drowning was assessed with regard to bathing (only if the child was bathed), safety around ponds (only if a pond was present), safety around private swimming pools (only if a swimming pool was present), and swimming (only if the child swam). Safety of bathing was assessed with 1 item: how often the child is left unsupervised in the bathtub, even for a short period (very often/often/sometimes/rarely/never). Safe bathing was defined as never leaving the child unsupervised in the bathtub. Safety of a pond was assessed with 1 item: presence of a fence around the pond (yes, fence higher than 1.20 m/yes, fence lower than 1.20 m/no). A safe pond was defined as having a fence higher than 1.20 m around the pond. Safety of a private swimming pool was assessed with 1 item: presence of a fence around the swimming pool (yes, fence higher than 1.20 m/yes, fence lower than 1.20 m/no). A safe private swimming pool was defined as having a fence higher than 1.20 m around the swimming pool. Swimming was assessed with 2 items: whether the child wears a flotation device (always/often/sometimes/rarely/never) and how often the child is left alone in the swimming pool (very often/often/sometimes/rarely/never). Safe swimming was defined as the child always wears a flotation device in the swimming pool and is never left alone in the swimming pool (either a private swimming pool or a small inflatable swimming pool).

Prevention of burns was assessed from hot water safety in the bath and shower (depending on what is present in the home), drinking hot fluids with child on parent’s lap, and safety during cooking. Presence of thermostatic-controlled taps was assessed with 2 items: does the hot water tap in the bath have a thermostatic-controlled tap (yes/no) and does the hot water tap in the shower have a thermostatic-controlled tap (yes/no). Safe hot water taps in bath/shower was defined as having a thermostatic-controlled tap present. Drinking hot fluids was assessed with 1 item: how often the parent drinks hot fluids with the child on their lap (always/often/sometimes/rarely/never). Safe drinking of hot fluids was defined as never drinking hot fluid with their child on their lap. Safe cooking was assessed with 4 items: presence of a stove guard (yes/no); child present in the kitchen during cooking (always/often/sometimes/rarely/never); use of rear burners during cooking (always/often/sometimes/rarely/never); and turning pan handles away during cooking (always/often/sometimes/rarely/never). Safe cooking was defined as a stove guard present, the child never in the kitchen during cooking, always using rear burners during cooking, and always turning panhandles away during cooking.

Subsequently, a total risk score was calculated for each parent by summing each specific parent’s safety behavior for the 4 topics assessed (according to allocated scores shown in Multimedia Appendix 5). A higher score indicated more unsafe behavior. When a situation was not applicable, a score of zero was assigned. A maximum score of 53 points could be obtained. The individual scores of the total risk score were based on previous literature [17] and expert consultation (Consumer Safety Institute, the Netherlands).

### Demographic Factors

Parents’ gender, age, educational level, employment status, and ethnicity were assessed in the baseline questionnaire. Educational level was categorized as high, intermediate, or low. High level was defined as higher professional education or academic higher education; intermediate level as senior secondary vocational education, senior general secondary education, or university preparatory education; and low educational level as preparatory secondary vocational education or lower [38]. Employment status was defined as unemployed if they did not have a part-time or a full-time job. Parents’ ethnicity was determined based on their own parents’ country of birth (grandparents of the infant). A parent was of Dutch ethnicity if both grandparents were born in the Netherlands. If one of the grandparents was born in another Western country, a parent was of other Western ethnicity. If both grandparents were born in another Western or non-Western country, ethnicity was determined by the grandmother’s country of birth [38].

Family situation, number of children, child’s gender, age, and ability to crawl or walk were reported. Family situation was defined as single parent, or living with child or children and other parent or caregiver. Number of children in the family was dichotomized as first child in the family or second child or more children in the family. Crawling of the child was assessed and defined as an infant’s ability to crawl on hands and knees and/or crawl on their tummy and/or shuffle on their bottom (yes/no).

### Parent Evaluation of the E-Health4Uth Home Safety Module

Parents in the E-Health4Uth home safety intervention were asked to evaluate the E-Health4Uth home safety intervention module immediately after completing the module. Unless stated otherwise, all evaluation items were assessed on a 5-point Likert scale ranging from totally disagree (=1) to totally agree (=5).

An objective measure of parents’ exposure to the intervention was obtained from the log-in data from the intervention registration, which stored information on parents’ use of the intervention, including receipt of the tailored safety advice and completion of an implementation-intention plan.

Parents’ evaluation of the E-Health4Uth home safety intervention was assessed immediately after receiving the tailored safety advice and formulating an implementation-intention plan, by using a Web-based evaluation form. Parents reported the following items on the evaluation forms: the reading of the Web-based tailored safety advice (read completely, read partly, or did not read their advice); whether they found the advice to be reliable, understandable, relevant, useful, and motivating to take action; their intention to change their behavior with regard to falls, poisoning, drowning, and burns (yes/no); whether it was easy or difficult to complete an implementation-intention plan (from very difficult to very easy); time needed to answer the questions and read the safety advice (in minutes); whether they perceived this time to be long or short (from very long to very short); the ease of use of the E-Health4Uth home safety intervention (from very difficult to very easy); and whether they perceived it as being a pleasant source of information (from very unpleasant to very pleasant). Furthermore, they rated the E-Health4Uth home safety intervention on a scale from 1 (most negative) to 10 (most positive).

### Parent and Health Care Professional Evaluation of the Well-Baby Visit

Parents and child health care professionals in the E-Health4Uth home safety intervention were asked to evaluate the well-baby visit, including the discussion of the tailored safety advice, immediately after the well-baby visit at approximately age 11 months.

Parents reported their satisfaction with the information they discussed at the well-baby visit, whether discussing the tailored safety advice was a valuable supplement to receiving tailored safety advice, the overall satisfaction with the well-baby visit, and they rated the well-baby visit on a scale from 1 (most negative) to 10 (most positive).

Child health care professionals reported the time they needed for the well-baby visit (in minutes); the time needed to discuss the safety at home (in minutes); whether they gave a safety information leaflet to the parent (yes/no); whether the tailored safety advice was present in the child’s dossier (yes/no); whether the tailored safety advice was brought to the well-baby visit by the parent (yes/no); and whether the tailored safety advice was discussed with the parent during the well-baby visit (yes/no). Furthermore, the evaluation assessed whether the tailored safety advice was useful to discuss safety at home during the well-baby visit, the satisfaction with the information given, and the overall satisfaction with the well-baby visit. Child health care professionals rated the well-baby visit on a scale from 1 (most negative) to 10 (most positive).

### Statistical Analyses

#### Intention-To-Treat Analysis

An intention-to-treat analysis was applied [39]. Parents who were randomly assigned to either the intervention condition or the control condition were analyzed as such, regardless of whether they received the intervention or not. Cases with complete data on outcomes at follow-up were analyzed on the effectiveness of the intervention compared to the control condition.

Descriptive statistics were used to describe the characteristics of parents, children, and housing in the two study conditions. Differences between the intervention and control condition, as measured at baseline, were tested with an independent-samples *t* test or the Mann-Whitney *U* test (continuous variables) and chi-square test (categorical variables).

#### Effect Evaluation

The effectiveness of the E-Health4Uth home safety intervention was studied by means of logistic regression analyses (for all specific safety behaviors) and linear regression analyses (for total risk score). Regression analyses were performed with unsafe behavior of total risk score as dependent variable and condition (E-Health4Uth home safety intervention condition vs control condition) as independent variables. All regression analyses were adjusted for demographic factors that showed a significant difference between the two study conditions at baseline (*P*<.05).

Subsequently, it was determined whether the number of children, parents’ educational level, and parents’ ethnicity moderated the effects of the E-Health4Uth home safety intervention on unsafe behavior. This was done by adding an interaction term (group × demographic factor) to the regression analysis. If these interaction terms were significant at *P*<.05, stratified analyses were conducted. Results with a *P* value <.05 were considered to be statistically significant. All analyses were performed using SPSS 20.0 (IBM Corp, Armonk, NY, USA).

## Results

### Participants

A total of 1409 parents of the 3147 initially invited provided informed consent and completed the baseline questionnaire with a response rate of 44.79% (Figure 1). A total of 26 parents were excluded because they completed the questionnaire twice for the same family (1 questionnaire was removed from the database at random), or they did not meet the inclusion criteria of child’s age ≤12 months. After completing the baseline questionnaire, 696 parents were allocated to the E-Health4Uth home safety condition and 687 parents to the control condition. A total of 1292 parents completed the follow-up questionnaire (dropout rate 6.60%). Dropout was higher among mothers with a low educational level, unemployed mothers, and parents of non-Western ethnicity (*P*<.05). No other differences were observed between parents who completed the follow-up questionnaire and parents who were lost to follow-up. A study population of 1292 parents and their child were used in the analyses. Table 1 shows the family, child, and housing characteristics of the participants in the two study conditions. Most participants were mothers (93.58%), mean age 32.06 (SD 4.63) years, 15.19% had a low educational level, 83.44% were employed, and 88.46% were of Dutch ethnicity. Father’s mean age was 34.51 (SD 5.17) years; 22.40% had a low educational level, 95.67% were employed, and 87.94% were of Dutch ethnicity. In the present study, 2.26% of families included a single parent and 48.14% had 1 child. Of all children, 51.32% were boys, mean age 7.21 (SD 1.07) months, and 33.98% could crawl and 0.47% could walk. A main staircase was present in 87.52% of the homes, 36.41% had a window a child could reach, 11.07% had a pond present, and 3.18% had a private swimming pool at the home.

**Table 1:** Descriptive characteristics of the study sample^a^ at baseline (N=1292).

Characteristics			Total N=1292	Intervention n=643	Control n=649	*P* value^b^
**Family characteristics**						
	Mother is respondent, n (%)		1209 (93.58)	597 (92.85)	612 (94.30)	.16
	**Parent age** ^c^					
		Mother’s age (years), mean (SD)	32.06 (4.63)	32.08 (4.60)	32.04 (4.67)	.92
		Mother’s age (years), range	19.00-48.00	20.00-48.00	19.00-47.00	
		Father’s age (years), mean (SD)	34.51 (5.17)	34.50 (5.14)	34.52 (5.21)	.96
		Father’s age (years), range	21.00-56.00	22.00-56.00	21.00-55.0	
	**Mother’s educational level, n (%)** ^d^					.26
		High	524 (40.62)	254 (39.56)	270 (41.67)	
		Intermediate	570 (44.19)	280 (43.61)	290 (44.75)	
		Low	196 (15.19)	108 (16.82)	88 (13.58)	
	**Father’s educational level, n (%)** ^e^					.17
		High	470 (36.69)	217 (34.17)	253 (39.16)	
		Intermediate	524 (40.91)	268 (42.20)	256 (39.63)	
		Low	287 (22.40)	150 (23.62)	137 (21.21)	
	**Parent employment, n (%)** ^f^					
		Mother is employed	1068 (83.44)	512 (80.25)	556 (86.60)	.002
		Father is employed	1214 (95.67)	599 (94.93)	615 (96.39)	.20
	**Mother’s ethnicity, n (%)** ^g^					.67
		Dutch	1142 (88.46)	570 (88.65)	572 (87.27)	
		Other Western	60 (4.65)	32 (4.98)	28 (4.32)	
		Non-Western	89 (6.89)	41 (6.38)	48 (7.41)	
	**Father’s ethnicity, n (%)** ^h^					.43
		Dutch	1130 (87.94)	566 (88.58)	564 (87.31)	
		Other Western	65 (5.06)	34 (5.32)	31 (4.80)	
		Non-Western	90 (7.00)	39 (6.10)	51 (7.89)	
	Single parent, n (%)^d^		29 (2.26)	13 (2.03)	16 (2.49)	.58
	First child in family, n (%)		622 (48.14)	317 (49.30)	302 (47.00)	.41
**Child characteristics** ^i^						
	Gender (boys), n (%)		663 (51.32)	317 (49.30)	346 (53.31)	.15
	Age (months), mean (SD)		7.21 (1.07)	7.26 (1.08)	7.17 (1.07)	.17
	Age (months), range		4.73-11.56	4.73-11.56	4.76-11.47	
	Child can crawl, n (%)		438 (33.98)	231 (35.93)	207 (32.04)	.14
	Child can walk, n (%)		6 (0.47)	3 (0.47)	3 (0.47)	.99
**Housing characteristics** ^j^						
	Main staircase present, n (%)		1129 (87.52)	564 (87.71)	565 (87.33)	.83
	Windows a child can reach, n (%)		470 (36.41)	241 (37.48)	229 (35.34)	.42
	Pond present, n (%)		127 (11.07)	65 (11.40)	62 (10.75)	.72
	Private swimming pool present, n (%)		41 (3.18)	23 (3.58)	18 (2.79)	.42

^a^Participants with complete data available at baseline and follow-up.

^b^Differences between intervention condition and control condition, as measured at baseline, tested with independent-samples *t* test (continuous variables) and chi-square test (categorical variables).

^c^Missing data: n=20

^d^Missing data: n=2

^e^Missing data: n=11

^f^Missing data (mother): n=12; missing data (father): n=23

^g^Missing data: n=1

^h^Missing data: n=7

^i^Missing data (crawl): n=3; missing data (walk): n=2

^j^Missing data (staircase): n=2; missing data (window): n=1; missing data (pond): n=5; missing data (pool): n=4

### E-Health4Uth Home Safety Intervention Effects

Because the proportion of employed mothers (86.60%) was significantly higher in the control condition compared to those in the intervention condition (80.25%, *P*=.002), regression analyses were adjusted for mother’s employment status.

Concerning the prevention of falls, parents in the intervention condition showed significantly less unsafe behavior at follow-up for the top of the staircase (23.91% vs 32.19%; OR 0.65, 95% CI 0.50-0.85), the bottom of the staircase (63.53% vs 71.94%; OR 0.69, 95% CI 0.53-0.88), and the top and bottom of the staircase (68.94% vs 78.28%; OR 0.62, 95% CI 0.48-0.81) compared to parents in the control condition (Tables 2 and 3).

For the prevention of poisoning, parents in the intervention condition showed significantly less unsafe behavior with regard to the storage of cleaning products (30.33% vs 39.91%; OR 0.67, 95% CI 0.53-0.85) compared to parents in the control condition. For the prevention of drowning, parents in the intervention condition showed significantly less unsafe behavior with regard to bathing of the child (23.46% vs 32.25%; OR 0.65, 95% CI 0.51-0.84) compared to parents in the control condition.

For the prevention of burns, parents in the intervention condition showed significantly less unsafe behavior with regard to drinking hot fluids (34.84% vs 41.73%; OR 0.76, 95% CI 0.61-0.96) and using rear hotplates on the stove (79.34% vs 85.27%; OR 0.67, 95% CI 0.50-0.90) compared to parents in the control condition. There were no significant differences with regard to other specific behaviors between the two study conditions.

From baseline to follow-up, the prevalence of unsafe behavior in bathing of the child increased in both the intervention (5.74%-23.46%) and the control condition (6.29%-32.25%). Furthermore, from baseline to follow-up, the prevalence of unsafe behavior for children present in the kitchen increased in both the intervention (64.27%-91.24%) and the control condition (64.81%-93.18%). All other unsafe behaviors showed a decrease between baseline and follow-up.

At follow-up, parents in the intervention condition had a significantly lower total risk score (mean 13.63, SD 6.12; range 1.00-33.00) compared to parents in the control condition (mean 15.34, SD 6.07; range 0.00-37.00; beta coefficient=–1.59, 95% CI –2.26 to –0.93).

Explorative interaction analyses showed no significant interactions between number of children, parents’ educational level, and parents’ ethnicity with the intervention and control condition on unsafe behavior; therefore, stratified analyses were not conducted.

**Table 2:** Descriptive statistics of parents’ child safety behavior as measured at baseline and at follow-up for intervention and control condition (N=1292).

Behavior		Baseline, %			Follow-up, %		
		Intervention condition n=643	Control condition n=649	*P* value^a^	Intervention condition n=643	Control condition n=649	*P* value^a^
**Falls** ^b^							
	Unsafe top of staircase^c^	407 (72.29)	404 (72.14)	.96	137 (23.91)	187 (32.19)	.002
	Unsafe bottom of staircase^c^	497 (88.12)	504 (89.52)	.46	364 (63.53)	418 (71.94)	.002
	Unsafe top and bottom of staircase^c^	510 (90.43)	516 (91.65)	.47	395 (68.94)	454 (78.28)	< .001
	Unsafe windows a child can reach^d^	139 (57.68)	125 (54.59)	.50	97 (46.86)	101 (51.53)	.35
**Poisoning**							
	Unsafe storage of cleaning products	387 (60.19)	401 (61.98)	.30	195 (30.33)	259 (39.91)	.001
	Unsafe storage of medicines	247 (38.41)	255 (39.41)	.90	193 (30.02)	221 (34.16)	.14
**Drowning** ^b^							
	Unsafe bathing of the child^e^	36 (5.74)	40 (6.29)	.68	141 (23.46)	198 (32.25)	.001
	Unsafe pond^f^	54 (88.52)	49 (81.67)	.29	41 (77.36)	41 (77.36)	.99
	Unsafe swimming pool^g^	19 (82.61)	15 (83.33)	.95	6 (50.00)	11 (78.57)	.13
	Unsafe swimming^h^	253 (64.38)	256 (65.14)	.82	288 (51.61)	299 (52.73)	.71
**Burns**							
	Unsafe hot water taps in bath/shower	188 (30.18)	176 (27.76)	.35	167 (26.09)	162 (25.16)	.70
	Unsafe drinking hot fluids	351 (54.67)	328 (50.85)	.17	224 (34.84)	270 (41.73)	.01
	Unsafe cooking (not using a stove guard)	628 (97.82)	619 (96.12)	.08	591 (92.49)	610 (94.57)	.13
	Unsafe cooking (child in kitchen)	412 (64.27)	418 (64.81)	.84	583 (91.24)	601 (93.18)	.19
	Unsafe cooking (not using rear burners on stove)	589 (91.89)	576 (89.86)	.21	507 (79.34)	550 (85.27)	.005
	Unsafe cooking (not turning pan handles away)	334 (52.11)	321 (50.00)	.45	174 (27.27)	183 (28.33)	.67

^a^Mann-Whitney *U* test for continuous outcome, chi-square test for binominal outcomes.

^b^Only when applicable, such as a staircase is present (n=1154), a window a child can reach is present (n=404), the child is bathed (n=1220), a pond is present (n=106), a private swimming pool is present (n=26), or the child swims (n=1134).

^c^Baseline intervention: n=564, baseline control: n=565; follow-up intervention: n=573, follow-up control: n=581.

^d^Baseline intervention: n=241, baseline control: n=229; follow-up intervention: n=207, follow-up control: n=197.

^e^Baseline intervention: n=629, baseline control: n=637; follow-up intervention: n=601, follow-up control: n=619.

^f^Baseline intervention: n=65, baseline control: n=62; follow-up intervention: n=53, follow-up control: n=53.

^g^Baseline intervention: n=23, baseline control: n=18; follow-up intervention: n=12, follow-up control: n=14.

^h^Baseline intervention: n=402, baseline control: n=395; follow-up intervention: n=564, follow-up control: n=570.

**Table 3:** Outcomes of logistic regression analyses and linear regression analysis of the effect of E-Health4Uth home safety intervention on unsafe behavior at follow-up, with control condition as reference (N=1292).

Behavior		OR (95% CI)^a^	*P* value
**Falls** ^b^			
	Top of staircase	0.65 (0.50, 0.85)	.001
	Bottom of staircase	0.69 (0.53, 0.88)	.003
	Top and bottom of staircase	0.62 (0.48, 0.81)	< .001
	Windows a child can reach	0.86 (0.58, 1.27)	.44
**Poisoning**			
	Storage of cleaning products	0.67 (0.53, 0.85)	.001
	Storage of medicines	0.88 (0.69, 1.12)	.30
**Drowning** ^b^			
	Bathing of the child	0.65 (0.51, 0.84)	.001
	Pond	1.12 (0.44, 2.83)	.82
	Private swimming pool	0.27 (0.05, 1.51)	.14
	Swimming	0.98 (0.77, 1.24)	.87
**Burns**			
	Hot water taps in bath/shower	1.05 (0.82, 1.35)	.73
	Drinking hot fluids	0.76 (0.61, 0.96)	.02
	Cooking (using a stove guard)	0.76 (0.48, 1.20)	.23
	Cooking (child in kitchen)	0.81 (0.54, 1.23)	.33
	Cooking (using rear burners)	0.67 (0.50, 0.90)	.008
	Cooking (turning pan handles away)	0.94 (0.74, 1.20)	.63

^a^Logistic regression analyses with unsafe behavior as dependent variable and group (intervention condition vs control condition) as independent variable, adjusted for mother’s employment status.

^b^Only when applicable, such as a staircase is present (n=1154), a window a child can reach is present (n=404), the child is bathed (n=1220), a pond is present (n=106), a private swimming pool is present (n=26), or the child swims (n=1134).

^c^Linear regression analyses with unsafe behavior as dependent variable and group (intervention condition vs control condition) as independent variable, adjusted for mother’s employment status.

### Parent Evaluation of the E-Health4Uth Home Safety Module

Of all parents in the intervention condition (n=643), 587 completed the E-Health4Uth home safety module (91.29%). The Web-based evaluation form of the E-Health4Uth home safety module was completed by 541 of 643 parents (84.14%) immediately after completing the E-Health4Uth home safety module (Table 4).

The Web-based evaluation forms showed that 72.07% (369/541) of parents had read the tailored safety advice completely, 24.41% (125/541) had read it partly, and 3.52% (18/541) had not read their advice. Parents evaluated the received tailored safety advice as being reliable (mean 4.19, SD 0.75), understandable (mean 4.36, SD 0.60), relevant (mean 3.53, SD 0.92), useful (mean 3.90, SD 0.77), and motivating to take action with regard to safety at home (mean 3.60, SD 0.90).

An implementation-intention plan was completed by 68.80% (322/541) of parents; a second implementation-intention plan was completed by 31.20% (146/541) of parents. Parents positively evaluated the ease of completing an implementation-intention plan for their own situation (mean 4.08, SD 0.79). Parents spent a mean time of 14.44 (SD 7.08) minutes to answer the questions and read the safety advice; they evaluated this as being a short time (mean 3.20, SD 0.56). Parents positively evaluated the use of the E-Health4Uth home safety intervention (mean 4.05, SD 0.62) and found the intervention to be a pleasant source of information (mean 3.67, SD 0.78). Parents rated the E-Health4Uth home safety intervention with a mean score of 7.28 (SD 1.14).

**Table 4:** Evaluation of the E-Health4Uth home safety module by parents in the intervention condition immediately after completing the module (n=541).

Subject		n (%)/mean (SD)
**Reading of the Web-based, tailored safety advice, n (%)**		
	Have read their advice completely	369 (72.07)
	Have read their advice partly	125 (24.41)
	Have not read their advice	18 (3.52)
**Tailored safety advice, mean (SD)**		
	The safety advice was reliable^a^	4.19 (0.75)
	The safety advice was understandable^a^	4.36 (0.60)
	The safety advice was relevant^a^	3.53 (0.92)
	The safety advice was useful^a^	3.90 (0.77)
	The safety advice motivated to take action^a^	3.60 (0.90)
**Implementation-intention plan**		
	Completed an implementation-intention plan, n (%)	322 (68.80)
	Completed a second implementation-intention plan, n (%)	146 (31.20)
	Was it easy to complete an implementation-intention plan?, mean (SD)^a^	4.08 (0.79)
**E-Health4Uth home safety intervention, mean (SD)**		
	Minutes spent to answer the questions and read the safety advice^a^	14.44 (7.08)
	Did you think that the time spent to answer the questions and read the safety advice was (very) long or (very) short?^a^	3.20 (0.56)
	Was the intervention easy to use?^a^	4.05 (0.62)
	Was the intervention a pleasant source of information?^a^	3.67 (0.78)
	Rating for the Web-based, tailored safety advice intervention^b^	7.28 (1.14)

^a^Scores on a 5-point Likert scale ranging from 1 (most negative) to 5 (most positive).

^b^Scores from 1 (most negative) to 10 (most positive).

### Parent and Health Care Professional Evaluation of the Well-Baby Visit

During the well-baby visit, the tailored safety advice was discussed with 48.9% of the parents, was not discussed with 18.9%, and for 32.2% of the parents it was unclear whether the advice was discussed because no evaluation form was available and child health care professionals could not recall whether or not they had discussed this advice with the parent.

Parents (n=196) and child health care professionals (n=238) completed written evaluation forms immediately after the well-baby visit at which the tailored safety advice was discussed with the parent (Table 5).

Parents had a positive evaluation of the information discussed during the well-baby visit (mean 4.38, SD 0.62), rated discussing the tailored safety advice as a valuable supplement (mean 3.82, SD 0.87), and were satisfied overall with the well-baby visit (mean 4.38, SD 0.62). Parents rated the well-baby visit, including discussing the tailored safety advice, with a mean score of 8.20 (SD 0.87).

Child health care professionals reported that the mean total time spent in the well-baby visit was 20.40 (SD 4.51) minutes, with a mean of 5.70 (SD 2.27) minutes used for discussing safety at home. In addition to receiving tailored safety advice, the generic safety information leaflet was given to 72.03% (170/238) of the parents. The tailored safety advice was present in 87.82% (209/238) of the child dossiers and it was brought to the well-baby visit by 21.61% (51/238) of parents. Child health care professionals positively evaluated the tailored safety advice with regard to its usefulness to discuss safety at home during the well-baby visit (mean 3.77, SD 0.77), were satisfied with the information given to parents (mean 3.98, SD 0.64), and had an overall satisfaction with the well-baby visit (mean 4.01, SD 0.62). They rated the well-baby visit, including discussing the tailored safety advice, with a mean score of 7.30 (SD 0.79).

**Table 5:** Evaluation of the well-baby visit including discussing the tailored safety advice by parents in the intervention condition (n=196) and health care professionals (n=238) immediately after the well-baby visit.

Subject		n (%)/mean (SD)
**Parents, mean (SD)**		
	Satisfaction with information discussed^a^	4.38 (0.62)
	Discussing the tailored safety advice was a valuable supplement to the tailored safety advice?^a^	3.82 (0.87)
	Overall satisfaction with the well-baby visit^a^	4.38 (0.62)
	Rating for the well-baby visit^b^	8.20 (0.87)
**Child health care professionals**		
	Total time for well-baby visit (min), mean (SD)	20.40 (4.51)
	Time for safety during well-baby visit (min), mean (SD)	5.70 (2.27)
	Safety information leaflet given to the parent, n (%)	170 (72.03)
	Tailored safety advice present in dossier, n (%)	209 (87.82)
	Tailored safety advice brought by parent, n (%)	51 (21.61)
	Was the tailored safety advice useful to discuss safety at home during the well-baby visit?, mean (SD)^a^	3.77 (0.77)
	Satisfaction with information given, mean (SD)^a^	3.98 (0.64)
	Overall satisfaction with the well-baby visit, mean (SD)^a^	4.01 (0.62)
	Rating for the well-baby visit, mean (SD)^b^	7.30 (0.79)

^a^Scores on a 5-point Likert scale ranging from 1 (most negative) to 5 (most positive).

^b^Scores from 1 (most negative) to 10 (most positive).

## Discussion

### Principal Findings

This study evaluated the effect of Web-based, tailored, safety advice combined with personal counseling on parents’ child safety behaviors. Compared to counseling with generic written materials, the E-Health4Uth home safety intervention appeared to be effective in promoting several relevant parents’ child safety behaviors. As hypothesized, parents in the intervention condition showed significantly less unsafe behavior with regard to safe staircases, storage of cleaning products, bathing, drinking hot fluids, and cooking compared to parents who received counseling with generic written safety information. There were no significant differences for other specific behaviors between the two study conditions. At follow-up, parents in the intervention condition also showed a significantly lower total risk score compared to parents in the control condition.

Parents in the intervention group were positive about the E-Health4Uth home safety module and its use in well-baby visits was positively evaluated by both parents and child health care professionals.

This study confirms the results of previous studies that showed that applying techniques of computer-tailored safety education in a primary care setting was effective in adopting safety behaviors of parents when compared to receiving generic safety advice [17,40]. The present study focused on total risk scores and also investigated the effects of the E-Health4Uth home safety intervention on specific safety behaviors. This approach allowed for clarification of which specific safety behaviors the intervention is or is not effective for. These insights may guide the development and evaluation of additional approaches to improve parental safety behavior. To our knowledge, this is the first experimental study on the effectiveness of computer-tailored education to change parents’ safety behaviors.

These results support the use of Web-based tailored methods to help increase the effectiveness of parental safety advice. For the prevention of poisoning, the E-Health4Uth home safety intervention was effective on the storage of cleaning products. Although we anticipated that the intervention would have a similar effect on the storage of medicines, no difference was found in the unsafe storage of medicines between the intervention and control condition. At baseline, the prevalence of unsafe storage of medicines was, in fact, lower than the prevalence of unsafe storage of cleaning products. It is possible that the content of the intervention did not sufficiently increase parents’ motivation to store their medicines in a more safe way.

The E-Health4Uth home safety intervention was not effective on behaviors for window safety, storage of medicines, ponds, swimming pools and swimming, hot water taps of bath/showers, and some items of burns prevention. It is possible that the intervention did not sufficiently address these specific determinants of safe behavior. An explanation why the E-Health4Uth home safety intervention showed no effect on the behavior for window safety, ponds, and swimming pools could be the low numbers of households that had a pond or swimming pool. This affects statistical power so the results for these behaviors should be interpreted with care.

Despite a lower prevalence in the E-Health4Uth intervention condition, some behaviors appeared to be more unsafe at follow-up compared to baseline (eg, bathing of the child and cooking with the child present in the kitchen). This result was found in both the intervention and the control condition, and might be because of the change in both age and development of the child between baseline and follow-up (Multimedia Appendix 6). For example, with increasing age parents might assume they can leave the child alone in the bathtub, or it may be more difficult for the parent to keep the child out of the kitchen. However, such behavior is not recommended and current safety advice (either generic or individually tailored) still seems suboptimal. Moreover, despite the decrease of unsafe behavior in the two study conditions between baseline and follow-up, the prevalence of many unsafe behaviors remained high. The prevalence of unsafe behavior for the top/bottom of staircases, ponds, swimming pools, and some items of burns prevention was still over 70%. This indicates that the content of the tailored safety advice for these behaviors needs to be improved.

When parents reported at follow-up (child age approximately 17 months) that their child swam, they reported leaving their child unsupervised in the swimming pool sometimes (0.81%), rarely (4.19%), and never (95.00%; data not shown). Despite the fact that most parents never left their child unsupervised in the swimming pool, 5% still left their child unsupervised, although these were very young children. These children are at risk of drowning and should never be left unsupervised because they do not have swimming skills yet.

This study used a tailored home safety intervention delivered in a primary care setting. Next to primary care settings, computer-tailored home safety information can be applied to other health care settings, such as emergency departments [41]. This study also shows that computer-tailored home safety information is effective in improving parents’ child safety behavior.

Parents and youth health care professionals are positive about the tailored safety advice and the use of the tailored safety advice in well-baby visits. On the other hand, the intervention effect may have been diluted by suboptimal uptake of the novel method by parents and/or health care workers.

The E-Health4Uth home safety intervention consists of a home safety assessment questionnaire, Web-based tailored safety advice, an implementation-intention plan, and discussing the tailored safety advice with the child health care professional. However, the evaluations of the child health care professionals showed that the tailored safety advice was discussed with only 48.9% of the parents.

Unfortunately, we only received evaluation forms from approximately one-third of the health care professionals, so it is unknown whether the health care professionals discussed the tailored safety advice with the other parents. According to the evaluation forms we did receive from the health care professionals, there was a considerable chance of not having discussed the tailored safety advice. Examining this issue as a possible confounder in the logistic regression models showed similar results of the effect of the intervention on parents’ child safety behaviors compared to our findings without adjustment for the discussion of the tailored advice (data not shown). The main reasons for not discussing the safety advice with the parents were that (1) the well-baby visit was made by another child health care professional from another well-baby clinic who was not familiar with the study, (2) parents indicated that discussing the tailored safety advice was not necessary, and (3) the tailored safety advice was not present in the child’s dossier. However, although the tailored safety advice was not discussed with approximately 50% of the parents and uptake among parents needs improvement, a positive effect on parents’ child safety behavior was shown.

In daily practice, all parents (in both the intervention or control condition) received care as usual: the generic safety information leaflet. Parents in the intervention condition received the E-Health4Uth home safety intervention, but could also receive the generic safety information leaflet as a part of usual care.

### Strengths and Limitations

Our focus on the effect of a tailored intervention on both specific parents’ child safety behaviors and on an overall safety risk score is a major strength of this study. Other strengths include the randomized controlled design, the large number of participants (N=1292) and the small number lost to follow-up: only 6.6% of the participants failed to complete the follow-up questionnaire. However, dropout was higher among mothers with a low educational level, unemployed mothers, and parents of non-Western ethnicity, which could affect the generalizability of the results. In addition, the participation rate was 45%.

We may have recruited parents who were more receptive to this way of providing safety education; in this case, this could have led to an overestimation of the intervention effect. On the other hand, the study population was a reasonable reflection of the general population in the Netherlands [42].

Because we had low numbers of missing data and participants lost to follow-up (6.60%), missing data was not imputed. Given these low numbers, it is not likely that missing data lead to loss of power of the study [43].

The intervention was developed for use on computers with connection to the Internet. The intervention was not tested for functionality on mobile phones and tablets. Perhaps in the near future when implementing the intervention, it could be made accessible on all mobile devices that have access to the Internet.

Receiving gift vouchers may cause recall bias because parents could expect to receive further incentives in the future. This may have positively biased total effectiveness for both the experimental and control condition although the magnitude of any effects would be small. Despite this possible recall bias, the E-Health4Uth intervention is effective in specific parents’ child safety behaviors compared to receiving the control condition.

A high percentage of youth health care organizations declined to participate in the study. Of the 26 youth health care organizations that were initially invited to participate in the study, 5 volunteered to take part in the study. The main reason why health care providers did not wish to participate was that they were already involved in 1 or more other studies.

Finally, the high prevalence of unsafe parental behaviors might even be an underestimation of the real child safety situation. Because the present study relied on self-report of safety behavior by parents, misclassification might have occurred if parents gave socially desirable answers in order to look good (ie, overstating their safe behavior) [44-46]. Furthermore, self-report can be subject to recall bias or inaccurate responses. We tried to minimize the occurrence of socially desirable answers by ensuring confidentiality of the questionnaires. Earlier validation studies showed that there is an acceptable agreement between parents reported safety behavior and observations in homes of the parent [46]. Future studies with smaller samples assessing specific behaviors could include the use of home observations.

### Implications and Future Research

Findings from this study support the use of a tailored education approach involving the provision of tailored safety information. The tailored safety information was found to be more effective than generic safety information in promoting preventive behavior. Providing tailored safety information before a visit to the well-baby clinic might be more efficient because parents and child health care professionals can better prepare for this visit in which safety at home is discussed [47-50]. Moreover, the parents receive information that is more specific because it is tailored to the personal situation of the parent [23]. However, because the prevalence of unsafe behavior remains relatively high, additional approaches to improve parental safety behavior need to be developed.

To improve parents’ child safety behaviors, various cognitions (eg, perceived self-efficacy, perceived response efficacy, perceived vulnerability, and perceived severity) could be addressed [51-53]. Changing these cognitions about injury prevention behavior could possibly lead to more safe behavior. More insight is needed into why the Web-based, tailored, safety advice intervention is effective for some parents and not for others. Perhaps different determinants are correlated with different safety behaviors.

It is possible that parents have different motivations for change for different injury mechanisms. This is supported by the finding that the intervention is not equally effective for all parents’ child safety behaviors. Therefore, this issue needs to be further explored.

Future studies should also investigate the effect of discussing the tailored safety advice during the well-baby clinic visit in a larger sample, as well as other approaches to increase the effectiveness of the E-Health4Uth intervention. Also, more insight is needed on the effect of the intervention among various subgroups (eg, based on ethnicity or educational level).

### Conclusions

Compared to counseling with generic written materials, the E-Health4Uth home safety advice combined with counseling is effective in promoting parents’ child safety behavior for safe staircases, storage of cleaning products, bathing, drinking hot fluids, and cooking. There were no significant differences for other specific behaviors between the two study conditions.

Parents were positive about the E-Health4Uth home safety module and its use in well-baby visits was positively evaluated by both parents and child health care professionals. The results of this study support the application of Web-based, tailored, safety advice for the prevention of unintentional injuries in the youth health care setting.

## Multimedia Appendix 1

Safety advice of the intervention based on safety behavior in and around the home.

## Multimedia Appendix 2

Sample page 1 of tailored safety advice.

## Multimedia Appendix 3

Sample page 2 of tailored safety advice.

## Multimedia Appendix 4

Sample page 3 of tailored safety advice.

## Multimedia Appendix 5

Risk scores assigned to injury safety behaviors.

## Multimedia Appendix 6

Child characteristics at baseline and follow-up divided in intervention condition and control condition.

## Multimedia Appendix 7

CONSORT-EHEALTH V1.6.2 checklist [54].
