# Supplementary material for: Effectiveness of Web-Based Tailored Advice on Parents’ Child Safety Behaviors: Randomized Controlled Trial
Source: J Med Internet Res. 2014 Jan 24;16(1):e17. doi: 10.2196/jmir.2521 (PMC3913924; doi:10.2196/jmir.2521)
Supplement: Supplementary file 1 [file jmir_v16i1e17_app1.pdf]

Appendix 1. Safety advice of the intervention based on safety behavior in and around the home.

Safety behavior concerning the prevention of	*Applicable if:*	*Reinforcement or NO advice when:*
**Falls**		
- Stair gate	- The house has a staircase which the child can reach	- A stair gate is present at the top and bottom of the staircase and is being used at all times
- Windows	- The house has windows a child can reach	- A window restrictor is present
**Poisoning**		
- Cleaning products	- Always	- Stored in a closet with a lock or higher than 1.50 meters
- Medicines	- Always	- Stored in a closet with a lock or higher than 1.50 meters
**Drowning**		
- Bath tub	- The child takes a bath	- Never left alone in the bath tub
- Pond	- There is a pond present	- A fence higher than 1.20 meters is present around the pond
- Private swimming pool	- There is a swimming pool present	- A fence higher than 1.20 meters is present around the swimming pool
- Swimming	- The child swims	- The child always wears a flotation device and is never left alone in the swimming pool
**Burns**		
- Hot water taps of bath/shower	- Always	- Thermostat-controlled tap present
- Hot fluids	- Always	- Child never on parents lap when drinking hot drinks
- Cooking	- Always	- A stove guard is present
- Cooking	- Always	- Child never in the kitchen when cooking
- Cooking	- Always	- Always using rear hotplates during cooking
- Cooking	- Always	- Always turning panhandles away during cooking
