# Supplementary figures and images for: Effectiveness of Web-Based Tailored Advice on Parents’ Child Safety Behaviors: Randomized Controlled Trial
Source: J Med Internet Res. 2014 Jan 24;16(1):e17. doi: 10.2196/jmir.2521 (PMC3913924; doi:10.2196/jmir.2521)

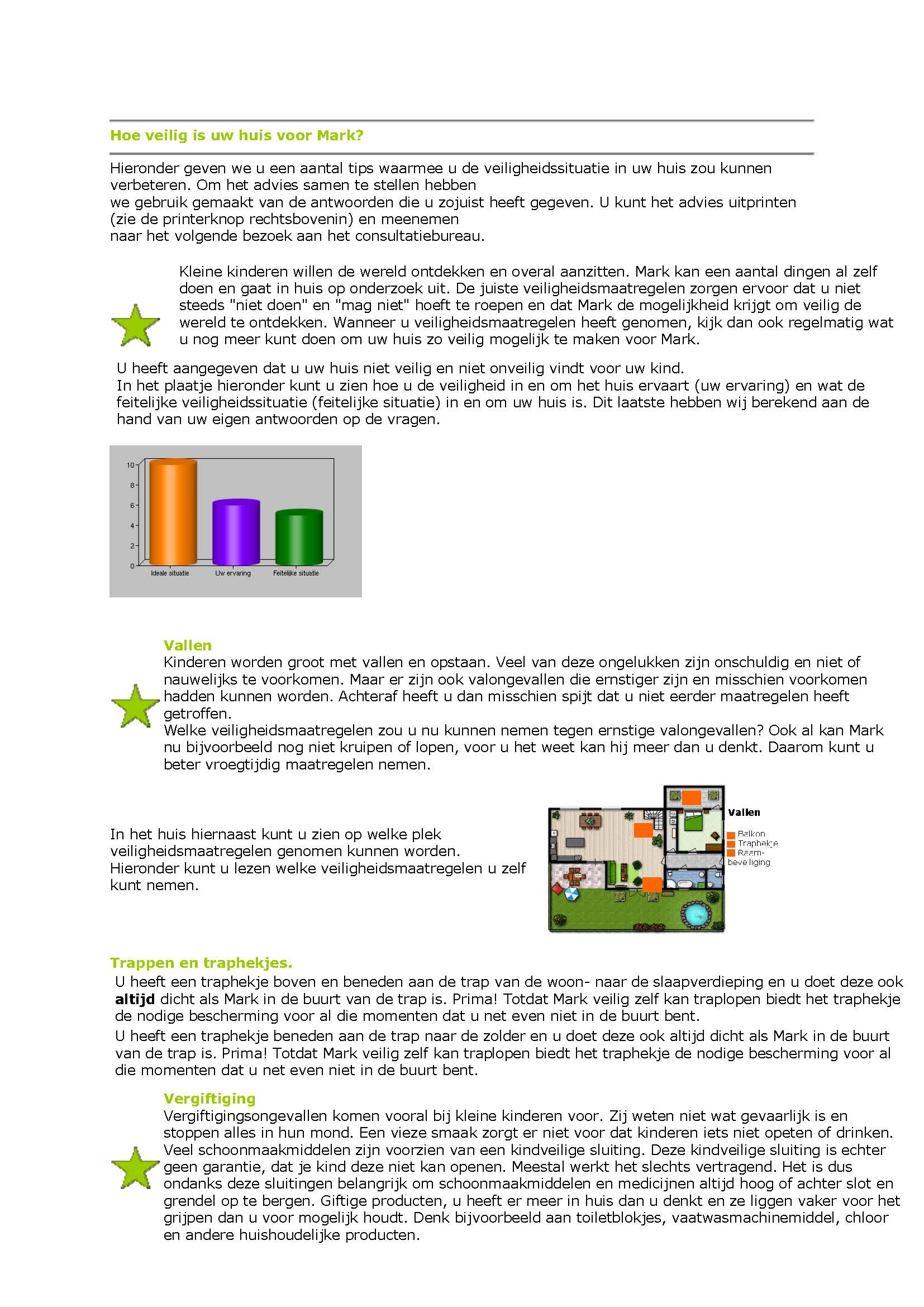

Supplement: Supplementary file 2 [file jmir_v16i1e17_app2.jpg]

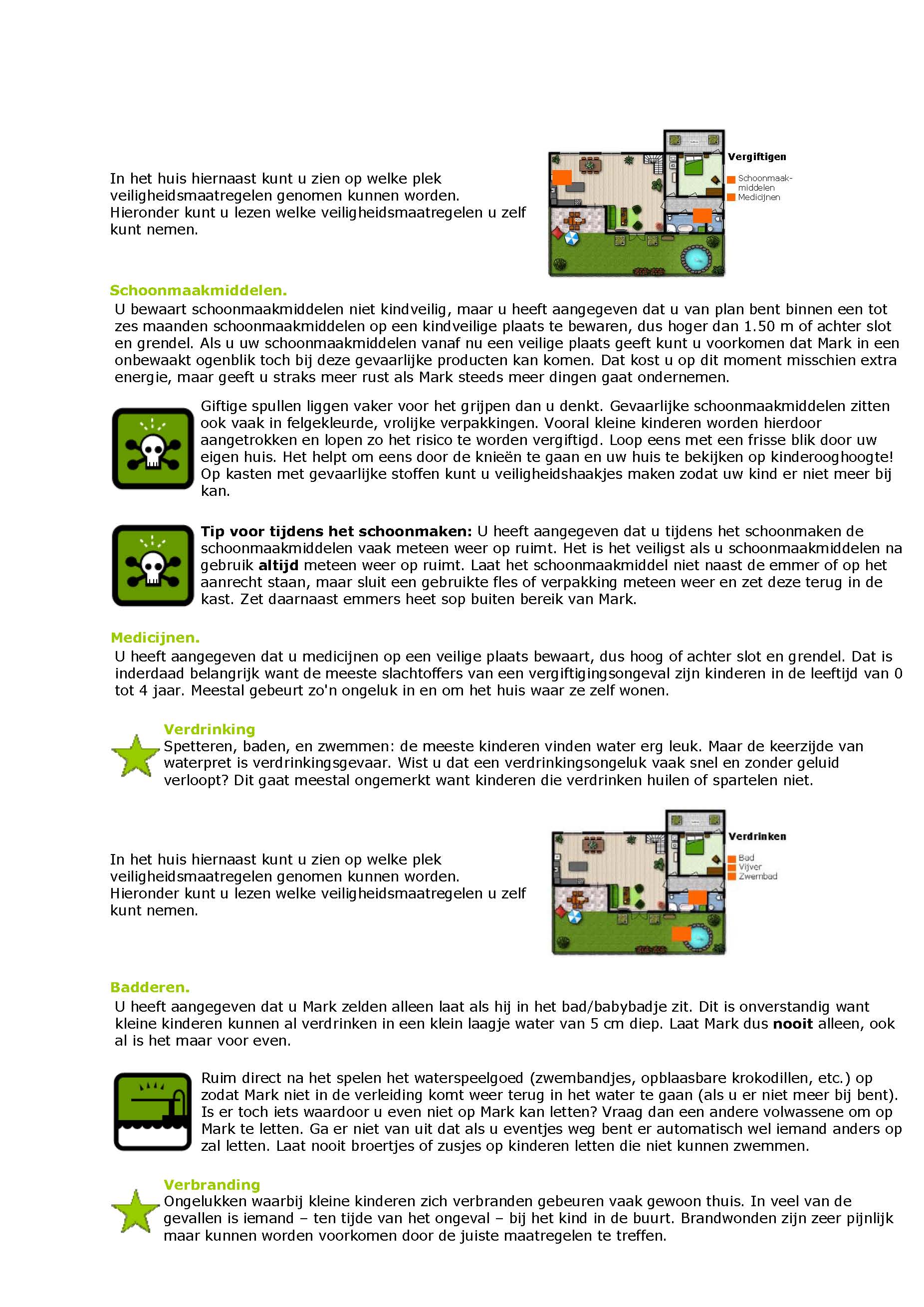

Supplement: Supplementary file 3 [file jmir_v16i1e17_app3.jpg]

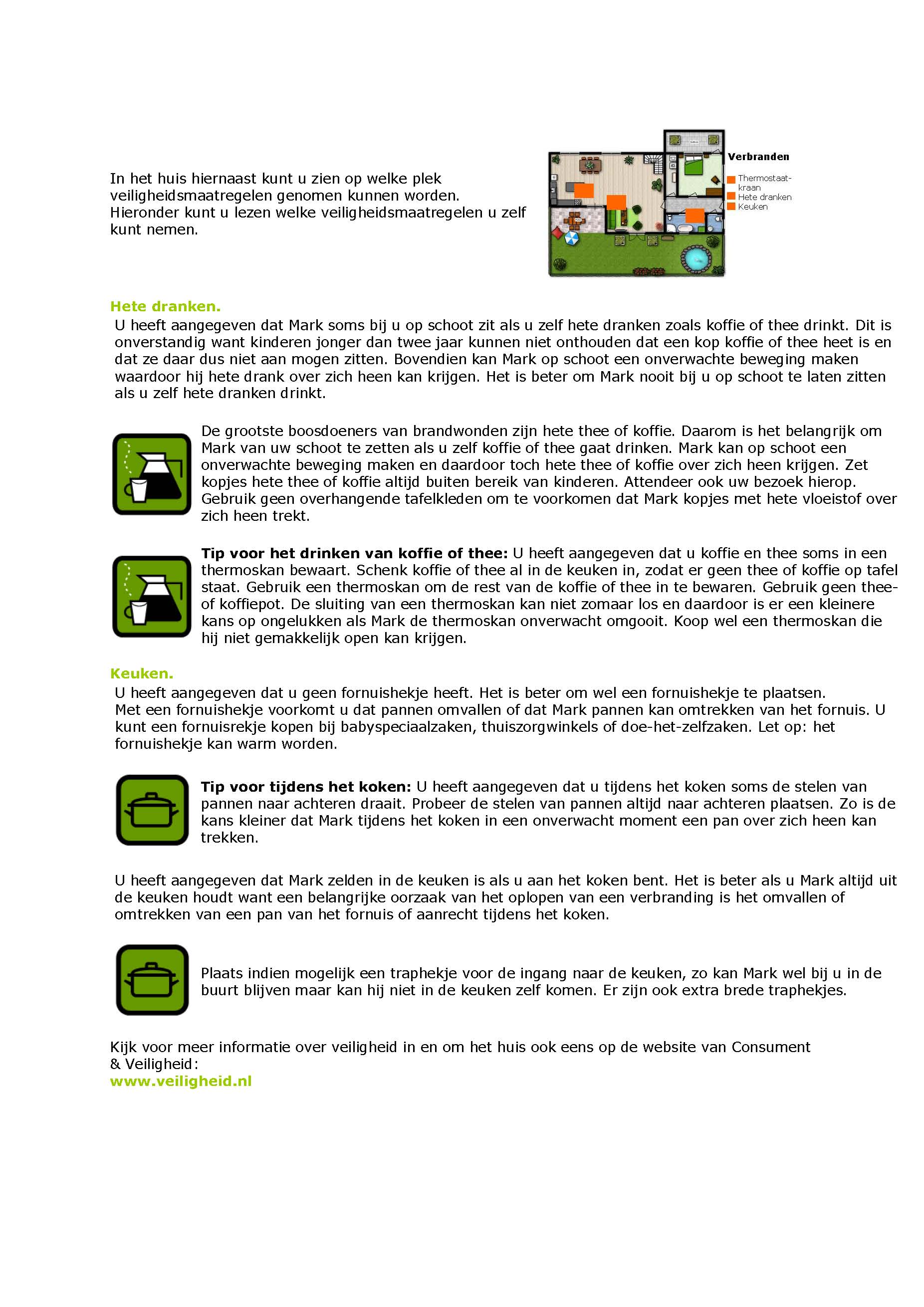

Supplement: Supplementary file 4 [file jmir_v16i1e17_app4.jpg]
