# Supplementary material for: Effectiveness of Web-Based Tailored Advice on Parents’ Child Safety Behaviors: Randomized Controlled Trial
Source: J Med Internet Res. 2014 Jan 24;16(1):e17. doi: 10.2196/jmir.2521 (PMC3913924; doi:10.2196/jmir.2521)
Supplement: Supplementary file 5 [file jmir_v16i1e17_app5.pdf]

Appendix 5. Risk scores* assigned to injury safety behaviors

	Injury safety behavior	Score
Falls	Stair gate present only at top or bottom of stairs	2
	No stair gate present	4
	Closing stair gate often	2
	Closing stair gate some of the time	3
	Rarely closing stair gate	4
	Never closing stair gate	4
	No window restrictor on windows a child can reach	2
Pois oning	Cleaning products stored unlocked above 1.50 m	1
	Cleaning products stored unlocked below 1.50 m	3
	Cleaning products stored on the floor	4
	Medicines stored unlocked above 1.50 m	1
	Medicines stored unlocked below 1.50 m	3
	Medicines stored on the floor	4
	Medicines stored in handbag	3
ingDrown	Child left alone in bath tub	5
	Pond present	3
	Pond with fence below 1.20 m	4
	Pond with no fence	5
	Pool with fence below 1.20 m	4
	Pool with no fence	5
	Often wearing (inflatable) flotation device when swimming	1
	Some of the time wearing (inflatable) flotation device when swimming	2
	Rarely wearing (inflatable) flotation device when swimming	2
	Never wearing (inflatable) flotation device when swimming	2
	Left alone in pool	5
Burns	No thermostatic controlled taps on hot water taps in bath or shower	4
	Child on parents' lap when drinking hot liquids	4
	Not using a stove guard	1
	Child in kitchen when parent is cooking	2
	Not using rear hotplates, when a stove guard is not present	1
	Not turning panhandles away from the front of the cooker, when a stove guard is not present	1

* Risk scores based on literature and expert consultation (Consumer Safety Institute, the Netherlands); when a situation is not applicable or safe, risk score is equal to 0. Higher scores indicate more unsafe behavior
