# Supplementary material for: Effectiveness of Web-Based Tailored Advice on Parents’ Child Safety Behaviors: Randomized Controlled Trial
Source: J Med Internet Res. 2014 Jan 24;16(1):e17. doi: 10.2196/jmir.2521 (PMC3913924; doi:10.2196/jmir.2521)
Supplement: Supplementary file 6 [file jmir_v16i1e17_app6.pdf]

Appendix 6. Child characteristics at baseline and follow-up divided in intervention condition and control condition (n=1292)

[missing data baseline-follow-up]	*Intervention condition* n=643	*Control condition* n=649	*P-value**
	Baseline		
Mean age (SD; range), months [8-0]	7.3 (1.1); 4.7-11.6	7.2 (1.1); 4.8-11.5	.17
Child can crawl (%) [0-3]	35.9	32.7	.14
Child can walk (%) [0-1]	0.4	0.4	.99
Lifetime prevalence of medically attended injury (%) [3-6]	3.0	2.7	.39
	Follow-up		
Mean age (SD; range), months [8-0]	16.8 (0.7); 15.8-22.5)	16.8 (0.8); 15.6-21.7)	.72
Child can crawl (%) [0-3]	98.6	9.2	.28
Child can walk (%) [0-1]	89.4	86.5	.12
Lifetime prevalence of medically attended injury (%) [3-6]	10.3	8.3	.30

* Differences between intervention condition and control condition, tested with independent-samples t-test (continuous variables) and Chi-square test (categorical variables)
