# Supplementary material for: Effectiveness of Web-Based Tailored Advice on Parents’ Child Safety Behaviors: Randomized Controlled Trial
Source: J Med Internet Res. 2014 Jan 24;16(1):e17. doi: 10.2196/jmir.2521 (PMC3913924; doi:10.2196/jmir.2521)
Supplement: Supplementary file 7 [file jmir_v16i1e17_app7.pdf]

CONSORT-EHEALTH Checklist V1.6.2 Report

(based on CONSORT-EHEALTH V1.6), available at [http://tinyurl.com/consort-ehealth-v1-6].

2521

Date completed

1/9/2013 4:41:16

by

Mirjam van Beelen

Effectiveness of Web-based, Tailored Advice on Parents' Child Safety Behaviors: A Randomized Controlled Trial

TITLE

1a-i) Identify the mode of delivery in the title

"Effectiveness of Web-based, Tailored Advice on Parents' Child Safety Behaviors: A Randomized Controlled Trial"

1a-ii) Non-web-based components or important co-interventions in title

"Effectiveness of Web-based, Tailored Advice on Parents' Child Safety Behaviors: A Randomized Controlled Trial"

1a-iii) Primary condition or target group in the title

"Effectiveness of Web-based, Tailored Advice on Parents' Child Safety Behaviors: A Randomized Controlled Trial"

ABSTRACT

1b-i) Key features/functionalities/components of the intervention and comparator in the METHODS section of the ABSTRACT

"Parents were randomly assigned to: 1) web-based, tailored safety advice combined with personal counseling (E-Health4Uth home safety intervention), or 2) usual care, i.e. counseling with generic written safety information leaflets (control condition). Parents in the intervention condition completed a web-based safety behavior assessment questionnaire, resulting in tailored safety advice which was discussed with their child healthcare professional at the well-baby visit (around 11 months of age). Parents in the control condition received counseling using generic safety information leaflets at the well-baby visit (around 11 months of age)."

1b-ii) Level of human involvement in the METHODS section of the ABSTRACT

"1) web-based, tailored safety advice combined with personal counseling (E-Health4Uth home safety intervention), or 2) usual care, i.e. counseling with generic written safety information leaflets (control condition). Parents in the intervention condition completed a web-based safety behavior assessment questionnaire, resulting in tailored safety advice which was discussed with their child healthcare professional at the well-baby visit (around 11 months of age). Parents in the control condition received counseling using generic safety information leaflets at the well-baby visit (around 11 months of age). Parents' child safety behaviors were derived from self-report questionnaires at baseline (7 months of age) and at follow-up (17 months of age)."

1b-iii) Open vs. closed, web-based (self-assessment) vs. face-to-face assessments in the METHODS section of the ABSTRACT

"1) web-based, tailored safety advice combined with personal counseling (E-Health4Uth home safety intervention), or 2) usual care, i.e. counseling with generic written safety information leaflets (control condition). Parents in the intervention condition completed a web-based safety behavior assessment questionnaire, resulting in tailored safety advice which was discussed with their child healthcare professional at the well-baby visit (around 11 months of age). Parents in the control condition received counseling using generic safety information leaflets at the well-baby visit (around 11 months of age). Parents' child safety behaviors were derived from self-report questionnaires at baseline (7 months of age) and at follow-up (17 months of age)."

1b-iv) RESULTS section in abstract must contain use data

"A total of 1292 parents (response rate 44.8%) were analyzed. At follow-up, parents in the intervention condition (n=643) showed significantly less unsafe behavior compared to parents in the control condition (n=649)"

1b-v) CONCLUSIONS/DISCUSSION in abstract for negative trials

"Compared to generic written materials, the E-Health4Uth home safety intervention seems more effective in promoting parents' safety behavior with regard to safe staircases, storage of cleaning products, bathing, drinking hot fluids, and cooking. This study supports the application of web-based, tailored safety advice for the prevention of unintentional injuries in the youth healthcare setting."

INTRODUCTION

2a-i) Problem and the type of system/solution

"In the Netherlands, all parents are invited to regularly attend (free of charge) scheduled visits at their well-baby clinic. During these visits, the growth and development of the child is monitored and relevant health information and vaccinations are provided. In the Netherlands, about 93% of parents attend one or more well-baby visits when their child is aged ≤ 4 years; the attendance rates range from about 50-93% between the specific age-related scheduled visits [10]. Parents receive health information on various topics, including nutrition, growth and child home safety [11]. Currently, this safety information is provided to parents using generic information leaflets which they receive at their regular visits to the well-baby clinic. Nevertheless, the required safety behavior of parents is often lacking, causing unnecessary risk of injury of young children [12-14].

To prevent other childhood disorders, the application of web-based, tailored tools (E-Health) has increased the effectiveness of health promotion effects [15-17]. E-Health is a broad, emerging field at the intersection of medical informatics, public health and business, referring to health services and information delivered or enhanced through the Internet and related technologies [18]. It involves the use of information and communications (especially the Internet) to improve or enable health and healthcare [19]. E-Health could be used to provide information for parents on several health topics, including home safety.

An E-health4Uth home safety intervention with web-based, tailored safety information was developed and applied. This concerns web-based, tailored safety information in combination with personal counseling at well-baby clinics on safety behaviors required for their child at home. A pilot study showed that most parents found this new internet-based, tailored safety information to be useful and applicable, and that the child healthcare professionals are enthusiastic about the E-health intervention [20]. However, no information is available about the effects of the new internet-based, tailored safety information on parents' child safety behaviors compared to the older method of safety education. Tailored information is thought to promote behavior change by providing personally-relevant feedback."

2a-ii) Scientific background, rationale: What is known about the (type of) system

"In the Netherlands, all parents are invited to regularly attend (free of charge) scheduled visits at their well-baby clinic. During these visits, the growth and development of the child is monitored and relevant health information and vaccinations are provided. In the Netherlands, about 93% of parents attend one or more well-baby visits when their child is aged ≤ 4 years; the attendance rates range from about 50-93% between the specific age-related scheduled visits [10]. Parents receive health information on various topics, including nutrition, growth and child home safety [11]. Currently, this safety information is provided to parents using generic information leaflets which they receive at their regular visits to the well-baby clinic. Nevertheless, the required safety behavior of parents is often lacking, causing unnecessary risk of injury of young children [12-14].
To prevent other childhood disorders, the application of web-based, tailored tools (E-Health) has increased the effectiveness of health promotion effects [15-17]. E-Health is a broad, emerging field at the intersection of medical informatics, public health and business, referring to health services and information delivered or enhanced through the Internet and related technologies [18]. It involves the use of information and communications (especially the Internet) to improve or enable health and healthcare [19]. E-Health could be used to provide information for parents on several health topics, including home safety.
An E-health4Uth home safety intervention with web-based, tailored safety information was developed and applied. This concerns web-based, tailored safety information in combination with personal counseling at well-baby clinics on safety behaviors required for their child at home. A pilot study showed that most parents found this new internet-based, tailored safety information to be useful and applicable, and that the child healthcare professionals are enthusiastic about the E-health intervention [20]. However, no information is available about the effects of the new internet-based, tailored safety information on parents' child safety behaviors compared to the older method of safety education. Tailored information is thought to promote behavior change by providing personally-relevant feedback."

METHODS

3a) CONSORT: Description of trial design (such as parallel, factorial) including allocation ratio

"This study evaluates the effect of web-based, tailored safety information combined with personal counseling on parents' child safety behaviors with regard to the prevention of falls, poisoning, drowning and burns. The hypothesis is that parents in the E-Health4Uth home safety intervention condition will show less unsafe behavior and will have a lower total risk score 6 months post-intervention, compared to parents in the control condition with usual care.
In addition, the use and application of the E-Health4Uth home safety module and the well-baby visit, including the use of the tailored safety advice, were evaluated."

3b) CONSORT: Important changes to methods after trial commencement (such as eligibility criteria), with reasons

Not applicable; there were no changes to the methods

3b-i) Bug fixes, Downtimes, Content Changes

Not applicable; there were no changes

4a) CONSORT: Eligibility criteria for participants

"All parents with a child aged 5-8 months (one parent per family) who were eligible for a routine well-baby visit at their well-baby clinic from June 2009 until December 2010 received written information about the study and were invited to provide informed consent to participate (n= 3147)."

4a-i) Computer / Internet literacy

"Parents could complete the E-Health4Uth home safety module wherever they wished (e.g. at home, at work, etc.)"

4a-ii) Open vs. closed, web-based vs. face-to-face assessments:

"Managers of an opportunity sample of 26 youth healthcare organizations in the Netherlands were informed about the study and invited to participate. Five youth healthcare organizations in the mixed urban-rural provinces of Zuid-Holland, Noord-Brabant and Zeeland volunteered to participate, with a total of 30 well-baby clinics.
All parents with a child aged 5-8 months (one parent per family) who were eligible for a routine well-baby visit at their well-baby clinic from June 2009 until December 2010 received written information about the study and were invited to provide informed consent to participate (n= 3147). Parents who provided informed consent were invited to complete the baseline questionnaire.
Subsequently, parents were randomly assigned to one of two conditions: 1) web-based, tailored safety advice module combined with discussion of the tailored safety advice at the well-baby visit (E-Health4Uth home safety intervention condition), or 2) care as usual, i.e. receiving a generic written safety information leaflet at the well-baby visit (control condition). Randomization was done using a computerized random allocation generator."

4a-iii) Information giving during recruitment

All parents with a child aged 5-8 months (one parent per family) who were eligible for a routine well-baby visit at their well-baby clinic from June 2009 until December 2010 received written information about the study and were invited to provide informed consent to participate (n= 3147). Parents who provided informed consent were invited to complete the baseline questionnaire.
"Subsequently, parents were randomly assigned to one of two conditions: 1) web-based, tailored safety advice module combined with discussion of the tailored safety advice at the well-baby visit (E-Health4Uth home safety intervention condition), or 2) care as usual, i.e. receiving a generic written safety information leaflet at the well-baby visit (control condition). Randomization was done using a computerized random allocation generator."

4b) CONSORT: Settings and locations where the data were collected

Managers of an opportunity sample of 26 youth healthcare organizations in the Netherlands were informed about the study and invited to participate. Five youth healthcare organizations in the mixed urban-rural provinces of Zuid-Holland, Noord-Brabant and Zeeland volunteered to participate, with a total of 30 well-baby clinics.
All parents with a child aged 5-8 months (one parent per family) who were eligible for a routine well-baby visit at their well-baby clinic from June 2009 until December 2010 received written information about the study and were invited to provide informed consent to participate (n= 3147). Parents who provided informed consent were invited to complete the baseline questionnaire.
"Subsequently, parents were randomly assigned to one of two conditions: 1) web-based, tailored safety advice module combined with discussion of the tailored safety advice at the well-baby visit (E-Health4Uth home safety intervention condition), or 2) care as usual, i.e. receiving a generic written safety information leaflet at the well-baby visit (control condition). Randomization was done using a computerized random allocation generator."

4b-i) Report if outcomes were (self-)assessed through online questionnaires

"Data on demographic factors and parents' child safety behaviors were collected at enrolment at about 7 months of age (baseline), and 6 months post-intervention at around 17 months of age (follow-up), by self-report questionnaires."

4b-ii) Report how institutional affiliations are displayed

Not applicable

5) CONSORT: Describe the interventions for each group with sufficient details to allow replication, including how and when they were actually administered

5-i) Mention names, credential, affiliations of the developers, sponsors, and owners

"The content and development of the E-Health4Uth home safety module was not changed during the study. Intervention software (TailorBuilder) was developed by OverNite Software Europe (OSE, Sittard, the Netherlands)."

5-ii) Describe the history/development process

"Therefore, an intervention with web-based, tailored safety advice combined with personal counseling (E-health4Uth home safety) was developed and applied."
"A pilot study showed that most parents found this new internet-based, tailored safety information to be useful and applicable, and that the child healthcare professionals are enthusiastic about the E-health intervention [20]."

5-iii) Revisions and updating
The intervention underwent no changes during evaluation process
5-iv) Quality assurance methods
Not applicable
5-v) Ensure replicability by publishing the source code, and/or providing screenshots/screen-capture video, and/or providing flowcharts of the algorithms used
In the appendices we added screenshots of the intervention.
5-vi) Digital preservation
In the appendices we added screenshots of the intervention.
5-vii) Access
"Parents were asked to complete the E-Health4Uth home safety module before their next routine well-baby visit at around 11 months of age. Parents could complete the E-Health4Uth home safety module wherever they wished (e.g. at home, at work, etc.) as long as internet was available."
5-viii) Mode of delivery, features/functionalities/components of the intervention and comparator, and the theoretical framework
"The E-Health4Uth home safety intervention aims at four major topics with regard to safety in/around the home of children aged 12-24 months, i.e. prevention of falls, poisoning, drowning and burns [5, 6, 14, 24-28]. The components of the four safety topics of the intervention are shown in Appendix 1.
Parents allocated to the E-Health4Uth home safety intervention condition received a personal login name and password by e-mail, when their child was around 10 months old. Parents were asked to complete the E-Health4Uth home safety module before their next routine well-baby visit at around 11 months of age. Parents could complete the E-Health4Uth home safety module wherever they wished (e.g. at home, at work, etc.) as long as internet was available. As a first step, parents completed a safety assessment questionnaire. The answers to this assessment questionnaire were used to generate a web-based, tailored safety advice, which parents could read immediately online. The tailored safety advice was personalized with the child's name, and consisted of messages tailored to the parent's current situation and safety behavior (Multimedia Appendices 2, 3, and 4). This included sections with general information on the importance and relevance of the injury area. A total of 114 messages, which could be combined in various ways based on the parent's answers to the assessment questionnaire, were developed for this tailored safety advice.
When parents had completed reading their personal safety advice, they were invited to formulate an implementation-intention plan. In this implementation-intention plan, parents planned specific actions, i.e. what, when and where, to improve their safety behavior and implement these in their home situation at a specified time [29, 30].
The tailored safety advice and implementation-intention plan of each parent was sent by e-mail to both the parent and the child healthcare professional, to prepare for the routine well-baby visit at age 11 months. At the well-baby visit, the child healthcare professional discussed the tailored safety advice and the implementation-intention plan with the parent, using motivational interviewing techniques [29-31]. About 4 weeks after the well-baby visit, parents received a reminder about their tailored safety advice and the implementation-intention plan by e-mail, to strengthen the message."
5-ix) Describe use parameters
"Parents were asked to complete the E-Health4Uth home safety module before their next routine well-baby visit at around 11 months of age."
5-x) Clarify the level of human involvement
"Parents allocated to the E-Health4Uth home safety intervention condition received a personal login name and password by e-mail, when their child was around 10 months old. Parents were asked to complete the E-Health4Uth home safety module before their next routine well-baby visit at around 11 months of age. Parents could complete the E-Health4Uth home safety module wherever they wished (e.g. at home, at work, etc.) as long as internet was available. As a first step, parents completed a safety assessment questionnaire. The answers to this assessment questionnaire were used to generate a web-based, tailored safety advice, which parents could read immediately online. The tailored safety advice was personalized with the child's name, and consisted of messages tailored to the parent's current situation and safety behavior (Multimedia Appendices 2, 3, and 4). This included sections with general information on the importance and relevance of the injury area. A total of 114 messages, which could be combined in various ways based on the parent's answers to the assessment questionnaire, were developed for this tailored safety advice.
When parents had completed reading their personal safety advice, they were invited to formulate an implementation-intention plan. In this implementation-intention plan, parents planned specific actions, i.e. what, when and where, to improve their safety behavior and implement these in their home situation at a specified time [29, 30].
The tailored safety advice and implementation-intention plan of each parent was sent by e-mail to both the parent and the child healthcare professional, to prepare for the routine well-baby visit at age 11 months. At the well-baby visit, the child healthcare professional discussed the tailored safety advice and the implementation-intention plan with the parent, using motivational interviewing techniques [29-31]. About 4 weeks after the well-baby visit, parents received a reminder about their tailored safety advice and the implementation-intention plan by e-mail, to strengthen the message."
5-xi) Report any prompts/reminders used
"Parents received a maximum of two reminders for completing the questionnaires. Parents who did not respond to the invitations to complete the follow-up questionnaire received a telephone call to motivate them to complete the intervention or the questionnaire. Parents in the intervention condition received a maximum of two reminders to complete the E-Health4Uth home safety module. If they did not respond, they received a telephone call to motivate them to complete the E-Health4Uth home safety module."
"About 4 weeks after the well-baby visit, parents received a reminder about their tailored safety advice and the implementation-intention plan by e-mail, to strengthen the message."
5-xii) Describe any co-interventions (incl. training/support)
Not applicable
6a) CONSORT: Completely defined pre-specified primary and secondary outcome measures, including how and when they were assessed
"Data on demographic factors and parents' child safety behaviors were collected at enrolment at about 7 months of age (baseline), and 6 months post-intervention at around 17 months of age (follow-up), by self-report questionnaires."
6a-i) Online questionnaires: describe if they were validated for online use and apply CHERRIES items to describe how the questionnaires were designed/deployed
Not applicable
6a-ii) Describe whether and how "use" (including intensity of use/dosage) was defined/measured/monitored
"An objective measure of parents' exposure to the intervention was obtained from the login data from the intervention registration, which stores information on parents' use of the intervention, including receiving the tailored safety advice and completion of an implementation-intention plan."
6a-iii) Describe whether, how, and when qualitative feedback from participants was obtained
"Parents' evaluation of the E-Health4Uth home safety intervention was assessed immediately after receiving the tailored safety advice and formulating an implementation-intention plan, using a web-based evaluation form."

"Parents and child healthcare professionals in the E-Health4Uth home safety intervention were asked to evaluate the well-baby visit, including the discussion of the tailored safety advice, immediately after the well-baby visit at around 11 months of age."
6b) CONSORT: Any changes to trial outcomes after the trial commenced, with reasons
Not applicable
7a) CONSORT: How sample size was determined

7a-i) Describe whether and how expected attrition was taken into account when calculating the sample size

Not applicable: was described in study protocol which was published online.

7b) CONSORT: When applicable, explanation of any interim analyses and stopping guidelines

Not applicable

8a) CONSORT: Method used to generate the random allocation sequence

"Subsequently, parents were randomly assigned to one of two conditions: 1) web-based, tailored safety advice module combined with discussion of the tailored safety advice at the well-baby visit (E-Health4Uth home safety intervention condition), or 2) care as usual, i.e. receiving a generic written safety information leaflet at the well-baby visit (control condition). Randomization was done using a computerized random allocation generator."

8b) CONSORT: Type of randomisation; details of any restriction (such as blocking and block size)

"Subsequently, parents were randomly assigned to one of two conditions: 1) web-based, tailored safety advice module combined with discussion of the tailored safety advice at the well-baby visit (E-Health4Uth home safety intervention condition), or 2) care as usual, i.e. receiving a generic written safety information leaflet at the well-baby visit (control condition). Randomization was done using a computerized random allocation generator."

9) CONSORT: Mechanism used to implement the random allocation sequence (such as sequentially numbered containers), describing any steps taken to conceal the sequence until interventions were assigned

"Subsequently, parents were randomly assigned to one of two conditions: 1) web-based, tailored safety advice module combined with discussion of the tailored safety advice at the well-baby visit (E-Health4Uth home safety intervention condition), or 2) care as usual, i.e. receiving a generic written safety information leaflet at the well-baby visit (control condition). Randomization was done using a computerized random allocation generator."

10) CONSORT: Who generated the random allocation sequence, who enrolled participants, and who assigned participants to interventions

"All parents with a child aged 5-8 months (one parent per family) who were eligible for a routine well-baby visit at their well-baby clinic from June 2009 until December 2010 received written information about the study and were invited to provide informed consent to participate (n= 3147).Parents who provided informed consent were invited to complete the baseline questionnaire.

Subsequently, parents were randomly assigned to one of two conditions: 1) web-based, tailored safety advice module combined with discussion of the tailored safety advice at the well-baby visit (E-Health4Uth home safety intervention condition), or 2) care as usual, i.e. receiving a generic written safety information leaflet at the well-baby visit (control condition). Randomization was done using a computerized random allocation generator."

11a) CONSORT: Blinding - If done, who was blinded after assignment to interventions (for example, participants, care providers, those assessing outcomes) and how

11a-i) Specify who was blinded, and who wasn't

Not applicable: Blinding was not possible

11a-ii) Discuss e.g., whether participants knew which intervention was the "intervention of interest" and which one was the "comparator"

Not applicable: Blinding was not possible

11b) CONSORT: If relevant, description of the similarity of interventions

"This study evaluated the effect of web-based, tailored safety advice combined with personal counseling on parents' child safety behaviors. Compared to counseling with generic written materials..."

12a) CONSORT: Statistical methods used to compare groups for primary and secondary outcomes

"An intention-to-treat analysis was applied [33]. Descriptive statistics were used to describe the characteristics of parents, children and housing in the two study conditions. Differences between the intervention and control condition, as measured at baseline, were tested with an independent samples t-test or the Mann-Whitney U-test (continuous variables) and Chi-square test (categorical variables).

Effect evaluation

The effectiveness of the E-Health4Uth home safety intervention was studied by means of logistic regression analyses (for all specific safety behaviors) and linear regression analyses (for total risk score). Regression analyses were performed with unsafe behavior of total risk score as dependent variable and condition (E-Health4Uth home safety intervention condition vs. control condition) as independent variables. All regression analyses were adjusted for demographic factors that showed a significant difference between the two study conditions at baseline ($P < .05$).

Subsequently, it was determined whether the number of children, parents' educational level and parents' ethnicity moderated the effects of the E-Health4Uth home safety intervention on unsafe behavior. This was done by adding an interaction term (group*demographic factor) to the regression analysis. If these interaction terms were significant at $P < .05$, stratified analyses were conducted."

12a-i) Imputation techniques to deal with attrition / missing values

"A total of 1292 parents completed the follow-up questionnaire (drop-out rate 6.6%). Drop-out was higher among mothers with a low educational level, unemployed mothers, and parents of non-Western ethnicity ($P < .05$). No other differences were observed between parents who completed the follow-up questionnaire and parents who were lost to follow-up.

A study population of 1292 parents and their child was used in the analyses."

12b) CONSORT: Methods for additional analyses, such as subgroup analyses and adjusted analyses

Not applicable

RESULTS

13a) CONSORT: For each group, the numbers of participants who were randomly assigned, received intended treatment, and were analysed for the primary outcome

"A total of 1409 parents provided informed consent and completed the baseline questionnaire; response rate 44.8% (Figure 1). A total of 26 parents were excluded because they completed the questionnaire twice for the same family (one questionnaire was at random removed from the database), or they did not meet the inclusion criteria (child's age ≤ 12 months). After completing the baseline questionnaire, 696 parents were allocated to the E-Health4Uth home safety condition and 687 parents to the control condition. A total of 1292 parents completed the follow-up questionnaire (drop-out rate 6.6%). Drop-out was higher among mothers with a low educational level, unemployed mothers, and parents of non-Western ethnicity ($P < .05$). No other differences were observed between parents who completed the follow-up questionnaire and parents who were lost to follow-up.

A study population of 1292 parents and their child was used in the analyses."

"Of all parents in the intervention condition, 587 completed the E-Health4Uth home safety module (91.3%). The web-based evaluation form, immediately after completing the E-Health4Uth home safety module, was completed by 541 parents (84.1%) (Table 4)."

"Parents (n=196) and child healthcare professionals (n=238) completed written evaluation forms immediately after the well-baby visit at which the tailored safety advice was discussed with the parent (Table 5)."

13b) CONSORT: For each group, losses and exclusions after randomisation, together with reasons

Data is also shown in a flow chart of participants.

"A total of 26 parents were excluded because they completed the questionnaire twice for the same family (one questionnaire was at random removed from the database), or they did not meet the inclusion criteria (child's age ≤ 12 months). After completing the baseline questionnaire, 696 parents were allocated to the E-Health4Uth home safety condition and 687 parents to the control condition. A total of 1292 parents completed the follow-up questionnaire (drop-out rate 6.6%)."

13b-i) Attrition diagram
A flow diagram of enrollment and follow-up is provided in the manuscript.

14a) CONSORT: Dates defining the periods of recruitment and follow-up
"All parents with a child aged 5-8 months (one parent per family) who were eligible for a routine well-baby visit at their well-baby clinic from June 2009 until December 2010 received written information about the study and were invited to provide informed consent to participate (n= 3147)."

"All parents received a follow-up questionnaire when their child was around 17 months old (6 months post-intervention). The baseline and follow-up data were collected from June 2009 until July 2011."
14a-i) Indicate if critical "secular events" fell into the study period
Not applicable
14b) CONSORT: Why the trial ended or was stopped (early)
Not applicable: The trial ended because all data was collected.
15) CONSORT: A table showing baseline demographic and clinical characteristics for each group
"Table 1 shows the family, child and housing characteristics of the participants in the two study conditions."
15-i) Report demographics associated with digital divide issues
"Most participants were mothers (93.6%); mean age 32.1 (SD 4.6) years; 15.2% had a low educational level; 83.4% was employed; and 88.5% was of Dutch ethnicity. Father's mean age was 34.5 (SD 5.2) years; 22.4% had a low educational level; 95.7% was employed; and 87.9% was of Dutch ethnicity. In the present study, 2.3% of families included a single parent; 48.1% had one child. Of all children, 51.3% were boys; mean age 7.2 (SD 1.1) months; 34.0% could crawl and 0.5% could walk."
16a) CONSORT: For each group, number of participants (denominator) included in each analysis and whether the analysis was by original assigned groups
16-i) Report multiple "denominators" and provide definitions
In the tables of the manuscript the specific number of participants for each group and each analysis was mentioned.

"An intention-to-treat analysis was applied"
16-ii) Primary analysis should be intent-to-treat
"An intention-to-treat analysis was applied"
17a) CONSORT: For each primary and secondary outcome, results for each group, and the estimated effect size and its precision (such as 95% confidence interval)
95% confidence intervals were used.
17a-i) Presentation of process outcomes such as metrics of use and intensity of use
"Evaluation of the E-Health4Uth home safety module, immediately after completing the module, by parents in the intervention condition
Parents in the E-Health4Uth home safety intervention were asked to evaluate the E-health4Uth home safety intervention module."

17b) CONSORT: For binary outcomes, presentation of both absolute and relative effect sizes is recommended
"Evaluation of the well-baby visit including discussing the tailored safety advice, immediately after the well-baby visit, by parents and child healthcare professionals in the intervention condition
Parents and child healthcare professionals in the E-Health4Uth home safety intervention were asked to evaluate the well-baby visit, including the discussion of the tailored safety advice, immediately after the well-baby visit at around 11 months of age."
18) CONSORT: Results of any other analyses performed, including subgroup analyses and adjusted analyses, distinguishing pre-specified from exploratory
Not applicable
18-i) Subgroup analysis of comparing only users
Not applicable: no subgroup analysis
19) CONSORT: All important harms or unintended effects in each group
Not applicable: no harms or unintended effects were found
19-i) Include privacy breaches, technical problems
Not applicable
19-ii) Include qualitative feedback from participants or observations from staff/researchers

"Evaluation of the E-Health4Uth home safety module, immediately after completing the module, by parents in the intervention condition
Of all parents in the intervention condition, 587 completed the E-Health4Uth home safety module (91.3%). The web-based evaluation form, immediately after completing the E-Health4Uth home safety module, was completed by 541 parents (84.1%) (Table 4).
The web-based evaluation forms show that 72.1% of parents had read the tailored safety advice completely, 24.4% had read it partly, and 3.5% had not read their advice.
Parents evaluated the received tailored safety advice as being reliable (mean 4.2, SD 0.8), understandable (mean 4.4, SD 0.6), relevant (mean 3.5, SD 0.9), useful (mean 3.9, SD 0.8), and motivating to take action with regard to safety at home (mean 3.6, SD 0.9).
An implementation-intention plan was completed by 86.5% of parents; a second implementation-intention plan was completed by 31.2% of parents. Parents positively evaluated the ease of completing an implementation-intention plan for their own situation (mean 4.1, SD 0.8). Parents spent a mean time of 14.4 (SD 7.1) min to answer the questions and read the safety advice; they evaluated this as being a short time (mean 3.2, SD 0.6). Parents positively evaluated the use of the E-Health4Uthe home safety intervention (mean 4.1, SD 0.6) and found the intervention to be a pleasant source of information (mean 3.7, SD 0.8). Parents rated the E-Health4Uth home safety intervention with a mean score of 7.3 (SD 1.1)."

"Evaluation of the well-baby visit including discussing the tailored safety advice, immediately after the well-baby visit, by parents and healthcare professionals in the intervention condition
During the well-baby visit, the tailored safety advice was discussed with 48.9% of the parents, was not discussed with 18.9%, and in 32.2% of the parents it was unclear whether the advice was discussed because no evaluation form was available and child healthcare professionals could not recall whether (or not) they had discussed this advice with the parent.
Parents (n=196) and child healthcare professionals (n=238) completed written evaluation forms immediately after the well-baby visit at which the tailored safety advice was discussed with the parent (Table 5).
Parents had a positive evaluation of the information discussed during the well-baby visit (mean 4.4, SD 0.6), rated discussing the tailored safety advice as a valuable supplement (mean 3.8, SD 0.9), and overall were satisfied with the well-baby visit (mean 4.4, SD 0.6). Parents rated the well-baby visit, including discussing the tailored safety advice, with a mean score of 8.2 (SD 0.9).
Child healthcare professionals reported that the mean total time spent for the well-baby visit was 20.4 (SD 4.5) min, with a mean of 5.7 (SD 2.3) min used for discussing the safety at home. In addition to receiving tailored safety advice, the generic safety information leaflet was given to 72.0% of the parents. The tailored safety advice was present in 87.8% of the child dossiers and it was brought to the well-baby visit by 21.6% of parents. Child healthcare professionals positively evaluated the tailored safety advice with regard to its usefulness to discuss safety at home during the well-baby visit (mean 3.8, SD 0.8); were satisfied with the information given to parents (mean 3.9, SD 0.6); and had an overall satisfaction with the well-baby visit (mean 4.0, SD 0.6). They rated the well-baby visit, including discussing the tailored safety advice, with a mean score of 7.3 (SD 0.8)."

DISCUSSION

20) CONSORT: Trial limitations, addressing sources of potential bias, imprecision, multiplicity of analyses

20-i) Typical limitations in ehealth trials

"Strengths and limitations
Our focus on the effect of a tailored intervention on both specific parents' child safety behaviors and on an overall safety risk score, is a major strength of this study. Other strengths include the randomized controlled design, the large number of participants (n=1292) and the small number lost to follow-up; only 6.6% of the participants failed to complete the follow-up questionnaire.
However, dropout was higher among mothers with a low educational level, unemployed mothers, and parents of non-Western ethnicity, which could affect the generalizability of the results. Moreover, because the participation rate was 45% w. We may have recruited parents who are more receptive to this way of providing safety education; in this case, this could have led to an overestimation of the intervention effect. On the other hand, the study population was a reasonable reflection of the general population in the Netherlands [34].

Finally, the high prevalence of unsafe parental behaviors might even be an underestimation of the real situation with regard to childhood safety. Because the present study relied on self-report of safety behavior by parents, misclassification might have occurred if parents gave socially desirable answers, i.e. overstating their safe behavior [35-37]."

21) CONSORT: Generalisability (external validity, applicability) of the trial findings

21-i) Generalizability to other populations

"Our focus on the effect of a tailored intervention on both specific parents' child safety behaviors and on an overall safety risk score, is a major strength of this study. Other strengths include the randomized controlled design, the large number of participants (n=1292) and the small number lost to follow-up; only 6.6% of the participants failed to complete the follow-up questionnaire.
However, dropout was higher among mothers with a low educational level, unemployed mothers, and parents of non-Western ethnicity, which could affect the generalizability of the results."

21-ii) Discuss if there were elements in the RCT that would be different in a routine application setting

"Findings from this study support the use of a tailored education approach involving the provision of tailored safety information. The tailored safety information was found to be more effective than generic safety information in promoting preventive behavior. Providing tailored safety information prior to a visit at the well-baby clinic might be more efficient, because parents and child healthcare professionals can better prepare for the visit in which safety at home is discussed [38-41]. Moreover, the parents receive more specific information because it is tailored to the personal situation of the parent [42]. However, because the prevalence of unsafe behavior remains relatively high, additional approaches to improve parental safety behavior need to be developed.
More insight is needed into why the web-based, tailored safety advice intervention is effective in some parents and not in others. Perhaps different determinants are correlated with different safety behaviors; future studies should focus on the highly-prevalent unsafe behaviors.
Future studies should also investigate the effect of discussing the tailored safety advice during the well-baby clinic visit, as well as other approaches to increase the effectiveness of the E-Health4Uth intervention. Also, more insight is needed on the effect of the intervention among various subgroups, e. g. based on ethnicity or educational level."

22) CONSORT: Interpretation consistent with results, balancing benefits and harms, and considering other relevant evidence

22-i) Restate study questions and summarize the answers suggested by the data, starting with primary outcomes and process outcomes (use)

"This study evaluated the effect of web-based, tailored safety advice combined with personal counseling on parents' child safety behaviors. Compared to counseling with generic written materials, the E-Health4Uth home safety intervention appeared to be effective in promoting several relevant parents' child safety behaviors. As hypothesized, parents in the intervention condition show significantly less unsafe behavior with regard to safe staircases, storage of cleaning products, bathing, drinking hot fluids, and cooking, compared to parents who received counseling with generic written safety information. At follow-up, parents in the intervention condition also showed a significantly lower total risk score compared to parents in the control condition. Parents were positive about the E-Health4Uth home safety module, and its use in well-baby visits was positively evaluated by both parents and child healthcare professionals."

22-ii) Highlight unanswered new questions, suggest future research

"Implications and future research
Findings from this study support the use of a tailored education approach involving the provision of tailored safety information. The tailored safety information was found to be more effective than generic safety information in promoting preventive behavior. Providing tailored safety information prior to a visit at the well-baby clinic might be more efficient, because parents and child healthcare professionals can better prepare for the visit in which safety at home is discussed [38-41]. Moreover, the parents receive more specific information because it is tailored to the personal situation of the parent [42]. However, because the prevalence of unsafe behavior remains relatively high, additional approaches to improve parental safety behavior need to be developed.
More insight is needed into why the web-based, tailored safety advice intervention is effective in some parents and not in others. Perhaps different determinants are correlated with different safety behaviors; future studies should focus on the highly-prevalent unsafe behaviors.
Future studies should also investigate the effect of discussing the tailored safety advice during the well-baby clinic visit, as well as other approaches to increase the effectiveness of the E-Health4Uth intervention. Also, more insight is needed on the effect of the intervention among various subgroups, e. g. based on ethnicity or educational level."

Other information

23) CONSORT: Registration number and name of trial registry

"Trial registration: Current Controlled Trials NTR1836"

24) CONSORT: Where the full trial protocol can be accessed, if available

"The ‘E-Health4Uth home safety’ study (BeSAFE study) is a randomized controlled trial with a baseline measure point prior to the intervention and a follow-up measure point 6 months after the intervention; the study is described in detail elsewhere [23]."

"23 . van Beelen ME, Beirens TM, Struijk MK, den Hertog P, Oenema A, van Beeck EF, et al. 'Besafe', Effect-Evaluation of Internet-Based, Tailored Safety Information Combined with Personal Counselling on Parents' Child Safety Behaviours: Study Design of a Randomized Controlled Trial. 2010; 10: 466. PMID: 20696070"

25) CONSORT: Sources of funding and other support (such as supply of drugs), role of funders

"Funding Source
This study is funded by a grant from ZonMw, the Netherlands Organization for Health Research and Development (project no. 50-50205-98-25028000). The study’s sponsors had no role in the design and conduct of the study, in the collection, analysis, and interpretation of the data, or in the preparation, review, approval, or submission of the manuscript."

X26-i) Comment on ethics committee approval

"The Medical Ethics Committee of the Erasmus Medical Center gave a ‘declaration of no objection’ for this study (MEC-2008-370)."

x26-ii) Outline informed consent procedures

"All parents with a child aged 5-8 months (one parent per family) who were eligible for a routine well-baby visit at their well-baby clinic from June 2009 until December 2010 received written information about the study and were invited to provide informed consent to participate (n= 3147). Parents who provided informed consent were invited to complete the baseline questionnaire."

X26-iii) Safety and security procedures

"Parents allocated to the E-Health4Uth home safety intervention condition received a personal login name and password by e-mail, when their child was around 10 months old."

X27-i) State the relation of the study team towards the system being evaluated

"Conflicts of interest
All authors declare that they have no competing interests, financial or otherwise, and they have no relation towards the system being evaluated."
